# Implications of Alternative Parameterizations in Structural Equation Models for Longitudinal Categorical Variables

**DOI:** 10.1017/psy.2024.23

**Published:** 2025-01-03

**Authors:** Silvia Bianconcini, Kenneth A. Bollen

**Affiliations:** 1 Department of Statistical Sciences, University of Bologna, Bologna, Italy; 2 Department of Psychology and Neuroscience, Department of Sociology, University of North Carolina, Chapel Hill, NC, USA

**Keywords:** autoregressive latent trajectory models, model equivalence, threshold invariance, underlying variables, variance constraints

## Abstract

When analyzing scaling conditions in latent variable structural equation models (SEMs) with continuous observed variables, analysts scaling a latent variable typically set the factor loading of one indicator to one and either set its intercept to zero or the mean of its latent variable to zero. When binary and ordinal observed variables are part of SEMs, the identification and scaling choices are more varied and multifaceted. Longitudinal data further complicate this. In SEM software, such as lavaan and Mplus, fixing the underlying variables’ variances or the error variances to one are two primary scaling conventions. As demonstrated in this paper, choosing between these constraints can significantly impact longitudinal analysis, affecting model fit, degrees of freedom, and assumptions about the dynamic process and error structure. We explore alternative parameterizations and conditions of model equivalence with categorical repeated measures.

Using data from the National Longitudinal Survey of Youth 1997, we empirically explore how different parameterizations lead to varying conclusions in longitudinal categorical analysis. More specifically, we provide insights into the specifications of the autoregressive latent trajectory model and its special cases—the linear growth curve and first-order autoregressive models—for categorical repeated measures. These findings have broader implications for a wide range of longitudinal models.

## 1 Introduction

Researchers increasingly use categorical endogenous observed variables in their structural equation models (SEMs). Binary and ordinal variables are among the most common. Examples include binary variables registering life events such as divorce, job loss, or pregnancy or ordinal variables such as general health status (e.g., poor, fair, good, very good, excellent). Although SEMs with and without such categorical endogenous variables share many similarities, there are differences. Identification issues, which are absent with continuous endogenous variables, arise with their categorical counterparts when assuming a continuous latent variable underlies the observed categorical response. This issue is particularly pronounced in the context of longitudinal data analysis, where the choice of constraints becomes pivotal. The common diagonally Weighted Least Squares with Mean and Variance adjustments (WLSMV) estimator, such as that in Mplus 8.6 (Muthén & Muthén, 1998–2017) and lavaan 0.6-16 (Rosseel, 2012),^1^ offers various techniques for estimating models with categorical data. Many textbooks and papers recommend imposing constraints on thresholds, errors and/or underlying variable variances to establish model identification when using WLSMV. This advice implies that researchers can apply these constraints almost automatically without a clear preference for one over the other. Our paper demonstrates that such constraints are not universally applicable, especially with longitudinal data. Different constraints can lead to alternative model specifications that are not always equivalent (Lee & Hershberger, 1990; Levy & Hancock, 2007; Raykov & Penev, 1999; Stelzl, 1986).

Furthermore, relying on default parameterizations in SEM software that set the variance of the normally distributed variable underlying the observed categorical response to one is not always appropriate. For instance, Grimm and Liu (2016) argue that this is inappropriate with linear growth curve models, and they suggest setting the error variances to one. While researchers agree that the choice of setting the variances of the underlying variable or its error variance to one does not impact the fit of factor models in cross-sectional applications (Kamata & Bauer, 2008; Muthén & Asparouhov, 2002; Paek et al., 2018; Wang et al., 2023), this interchangeability need not carry over to repeated measures. Several papers empirically compare the two specifications for linear latent growth models but arrive at contrasting conclusions. For Grimm and Liu (2016) and Lee et al. (2018), parameterization affects both model fit and parameter estimation because different assumptions about the dynamic process and its errors are embedded in the chosen specification. On the other hand, for Newsom and Smith (2020), the choice between these two parameterizations is otherwise arbitrary as they are equivalent, and one solution can be transformed into another.

Various authors have proposed alternative parameterizations for latent variable SEMs for longitudinal data, mainly focusing on linear growth models. Muthén and Asparouhov (2002) and Grimm and Liu (2016) propose to allow the means and variances of the underlying variables to be freely estimated except on the first occasion. Thresholds are kept invariant over time, but for a multi-group confirmatory factor model, Millsap and Yun-Tein (2004) relaxed this assumption for all but two thresholds. Mehta et al. (2004) propose a different parameterization for the linear growth model applied to categorical data. It consists of freely estimating all the latent response means and variances and fixing the first two thresholds to zero and one, respectively, keeping the remaining thresholds invariant over time. Prior to their work, Jöreskog (2001) relaxed the threshold invariance condition in this parameterization.

Taken together, we find conflicting advice on the identifying constraints to impose when analyzing longitudinal data with endogenous categorical variables and incompatible claims on the equivalency of the constraints. The result is confusion and possible misapplication of categorical SEM techniques.

This paper examines the technical aspects of parameterization and modeling in analyzing binary and ordered categorical repeated measures. We provide a clear and easily accessible summary of the closed-form relations between longitudinal model specifications under alternative parameterizations. The role of these parameterizations is examined for the Autoregressive Latent Trajectory (ALT) model introduced by Bollen and Curran (2004) for continuous outcomes and its special cases, the linear latent growth curve and the autoregressive model. Unlike existing research, our study provides a comprehensive overview of possible parameterizations, evaluating necessary and sufficient conditions for model equivalence. Drawing on established comparisons, we determine when different models are empirically indistinguishable, shedding light on the practical implications of choosing specific constraints in the analysis of longitudinal categorical data.

## 2 Motivating data and example

The autoregressive latent trajectory model has proven invaluable for exploring longitudinal dynamics, especially with regard to changes in multiple variables over time. This is evidenced by its application to a diverse range of phenomena, including developmental trajectories of anxiety and depression (Connell et al., 2021; Lee & Vaillancourt, 2020; McLaughlin & King, 2015), trends in disposition and happiness (Caprara et al., 2017), and between aggressive behaviors and peer victimization in preadolescence (Yao & Enright, 2022). In the literature, it has been compared with other models that incorporate lagged effects alongside random components, such as the Random-Intercept Cross-Lagged Panel Model (RI-CLPM) (Hamaker et al., 2015) and the general cross-lagged panel model (Zyphur et al., 2020). A comprehensive discussion can be found in Usami (2021). A recent study by Andersen (2022) has also clarified and proven that the RI-CLPM is a constrained version of the ALT model.

While the model has been extensively applied to continuous variables, its application to categorical variables remains underexplored in the literature. This paper addresses this gap by presenting alternative specifications of the ALT model and two special cases—the linear growth curve and autoregressive of order one model—tailored for scenarios involving categorical variables.

To illustrate the practical implications of our study, we turn to a concrete example examining the co-occurrence of illegal drug use, depressive symptoms, and general health over time. Previous research by Silver et al. (2023) touched upon this nexus but applied default lavaan parameterization, treating illegal drug use as binary and depressive symptoms and general health as continuous variables. They separately analyzed autoregressive and cross-lagged effects and multivariate latent growth patterns for the three variables. Utilizing data from the National Longitudinal Survey of Youth 1997 (NLSY97), our study focuses on repeated measures of these variables as respondents transition from adolescence to adulthood, covering waves from 2000 to 2010. NLSY97 is a national study of 8984 respondents born in the US between January 1, 1980, and December 31, 1984. The respondents participated in in-person or phone interviews annually from 1997 (wave 1) to 2013 (wave 16), with two additional interviews in 2015 (wave 17) and 2017 (wave 18). For information on NLSY97 sampling and interviewing methods, refer to Moore et al. (2000).

Due to the lack of consensus on how to handle missing data with categorical variables, we followed the default option in lavaan 0.6-16 and applied listwise deletion, reducing the sample from 8,984 to 5,309 cases.^2^ Participants’ age ranged from 13 to 17 (average age: 14.92) during wave 4 and from 23 to 27 during wave 14. Data were analyzed for 2000 (wave 4), 2002 (wave 6), 2004 (wave 8), 2006 (wave 10), 2008 (wave 12), and 2010 (wave 14).

Illegal drug use was assessed by a single item asking respondents if they had used any illegal drugs (excluding marijuana and alcohol) since the previous interview. Responses were coded as one if the respondent had used illegal drugs and zero if he/she had not.

Depressive symptoms were evaluated based on respondents’ answer to the question: “*How much of the time during the last month have you felt so down in the dumps that nothing could cheer you up?*”. Ordinal responses were “all of the time,” “most of the time,” “some of the time,” and “none of the time,” with higher scores indicating more frequent depressive symptoms.

General health status was self-reported by respondents in answer to the question: *“In general, how is your health?”*. Choices were “excellent,” “very good,” “fair,” and “poor.” The measures of general health were coded with higher scores, indicating better perceived general health.

Table 1 presents the marginal proportions for each category of the observed variables at each time point. The frequency of adolescents experiencing depressive symptoms indicates a decrease in the likelihood of being more depressed as they age into adulthood. Similarly, the propensity to use illegal drugs and the perception of excellent health decreases over time, while the perception of good and very good health remains relatively constant, especially in the adulthood period (waves 10–14).

**Table 1:** Proportions for each year of the categories related to depressive symptoms, general health status, and illegal drug use

Wave (year)	Depressive symptoms				Illegal drug use	
	None of the time	Some of the time	Most of the time	All the time	No	Yes
4 (2000)	0.637	0.297	0.054	0.012	0.930	0.070
6 (2002)	0.639	0.296	0.052	0.014	0.937	0.063
8 (2004)	0.687	0.262	0.043	0.008	0.943	0.057
10 (2006)	0.721	0.238	0.035	0.007	0.950	0.050
12 (2008)	0.718	0.242	0.032	0.008	0.960	0.040
14 (2010)	0.732	0.230	0.030	0.008	0.968	0.032
Wave (year)	General health status					
	Poor	Fair	Good	Very good	Excellent	
4 (2000)	0.006	0.052	0.241	0.330	0.371	
6 (2002)	0.006	0.061	0.254	0.369	0.311	
8 (2004)	0.005	0.065	0.264	0.371	0.295	
10 (2006)	0.007	0.070	0.271	0.373	0.280	
12 (2008)	0.008	0.076	0.298	0.375	0.244	
14 (2010)	0.013	0.094	0.293	0.368	0.232	

In contrast to conventional approaches, we explore various specifications of the ALT model, considering different constraints on thresholds, means of the underlying variables, and error variances. Our findings reveal that the choice of these identification constraints significantly influences the results, challenging the notion of interchangeable specifications.

**Figure 1:**
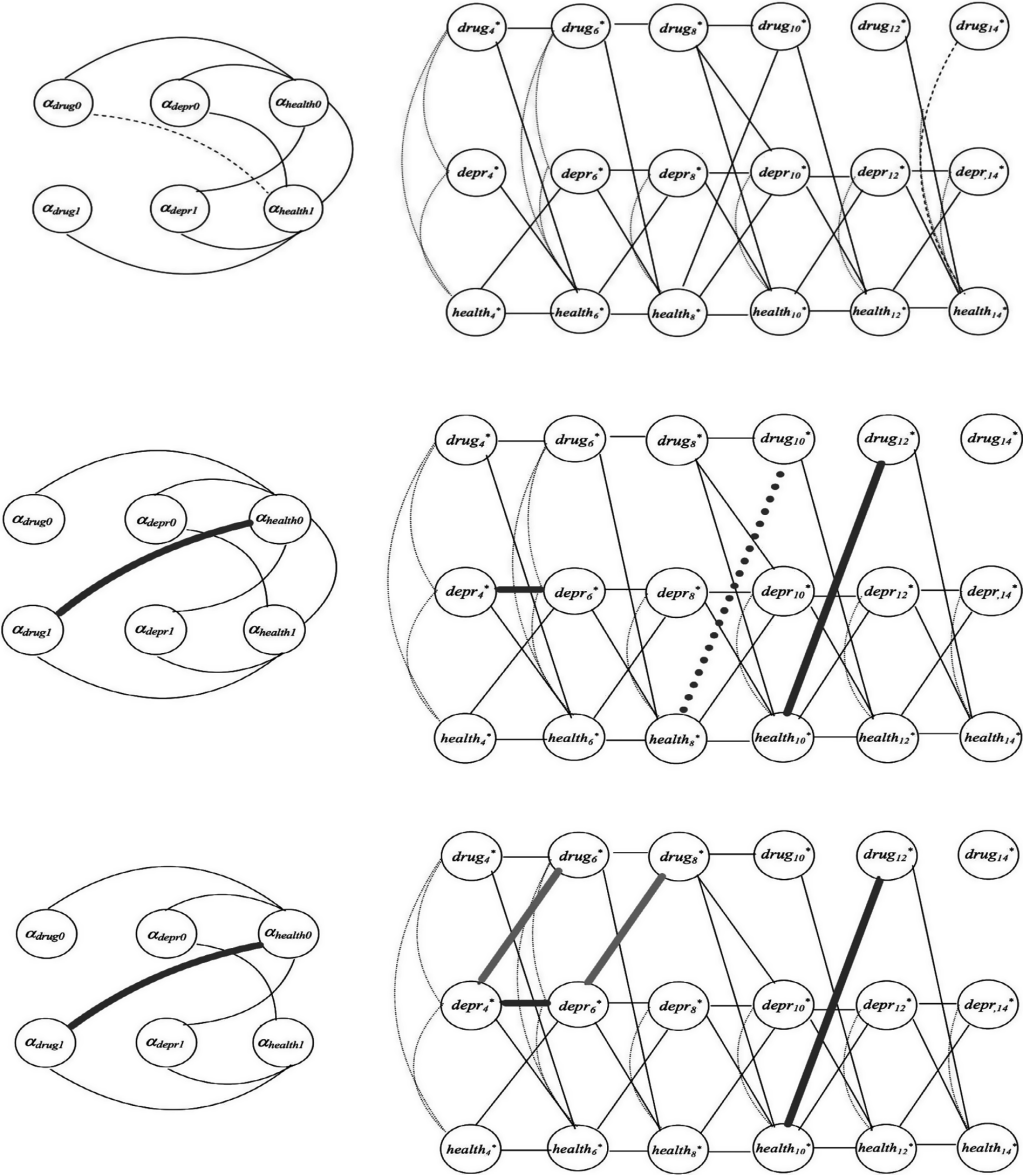
Path diagram depicting the cross-lagged and autoregressive component (*right*) and multivariate growth part (*left*) of alternative autoregressive latent trajectory model specifications for illegal drug use (*drug*), depressive symptoms (*depr*), and general health status (*health*). The top panel is based on the standard (theta) parameterization of the auxiliary model linking observed variables to the underlying continuous ones, as adopted by Mplus 8.6 and lavaan 0.6-16. Dashed lines indicate statistically significant effects ( 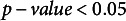 ) only under this specification. The middle panel showcases an alternative parameterization proposed by Muthén and Asparouhov (2002). Paths in dark gray signify significance under the alternative parameterizations but not under the standard one. The bottom panel employs the parameterization introduced by Jöreskog (2001). Light gray paths indicate a significant pattern under this specification but not in the others.

Figure 1 illustrates different estimated ALT specifications, demonstrating the impact of different parameterizations on cross-lagged and autoregressive relationships (on the right) as well as growth components (on the left). Only statistically significant 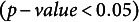 path coefficients are presented for simplicity. The two ALT components—the multivariate growth and the cross-lagged and autoregressive part—are shown separately for illustrative purposes, although they are estimated simultaneously to describe the temporal dynamic of the underlying latent variables.

The upper panel in Figure 1 adopts the standard (theta) parameterization of Mplus 8.6 and lavaan 0.6-16. In this configuration, all the error variances are set to one, all the thresholds in the model linking observed variables to the underlying continuous ones are freely estimated, and the mean of the underlying variables is fixed to zero.

Regarding autoregressive and cross-lagged relationships, prior illegal drug use, depressive symptoms, and previous health status perception consistently impact general health status on each occasion. However, only depressive symptoms and health status on the preceding occasion influence the propensity to experience depression on each occasion.

Concerning illegal drug use, no significant cross-lagged effects are observed; instead, there is a direct effect of the illegal drug use propensity on the preceding occasion on subsequent illegal drug use propensity in the initial three waves (4, 6, and 8 - corresponding to the years 2000, 2002, and 2004), associated with late adolescence/early adulthood. This contrasts with the middle panel in Figure 1, where a significant influence of general health status at waves 8 and 10 on illegal drug use propensities in waves 10 and 12 (associated with the years 2006 and 2008), respectively, is observed. This ALT specification uses an alternative parameterization proposed by Muthén and Asparouhov (2002) for categorical longitudinal data. In this approach, instead of fixing the error variances to one on all occasions, they are freely estimated on all occasions but one, with thresholds assumed to be time-invariant.

Conversely, in the lower panel of Figure 1, the propensities for illegal drug use during waves associated with late adolescence (6 and 8) are significantly influenced by depressive symptoms in the preceding occasion. This influence is specific to the late adolescence/early adulthood phase and not applicable during adulthood. The model parameterization in this lower panel aligns with the alternative approach proposed by Jöreskog (2001). It involves freely estimating the error variances and the means of the underlying variables on each occasion while fixing the first two thresholds to zero and one on all occasions.

The impact of alternative parameterizations of the ALT model is also noticeable in the estimated multivariate growth component. In the standard parameterization (top left panel), a correlation is observed between the intercept for the illegal drug use variable and the slope of general health status. Conversely, under the alternative parameterizations (middle and bottom panels), a correlation is estimated between the slope of illegal drug use propensity and the intercept of the growth component for health status perception. Notably, in the bottom panel, there is no significant covariance between the intercept and slope growth factors for general health status, distinguishing it from other specifications.

These findings highlight the critical importance of carefully selecting the most suitable parameterization for models involving categorical longitudinal data. The following sections offer a theoretical interpretation and practical application of these results.

## The auxiliary measurement model linking 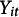 and 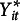


3

Let 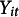 be the ordered categorical measure for the *i*th person on the *t*th occasion, with 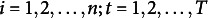 . The values of 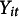 range from 0 to 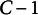 , where *C* is the number of response categories across all occasions.

In SEMs, a common approach is to consider the observed values of 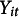 as discretized manifestations of an underlying continuous variable, denoted as 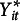 . Depending on the measure, this underlying variable represents the level of understanding, attitude, or propensity to respond in a particular category. In our application on the NLSY data, it reflects the propensity to respond for all three variables under investigation. The observed category is then determined through 
(1)

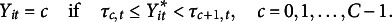

Here, 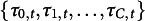 are threshold parameters for 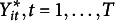 , where two of them are predefined, 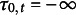 and 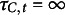 , whereas the remaining 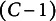 may vary across occasions. Let 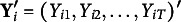 be a *T*-dimensional vector representing the observed scores for the *i*th person on the *T* occasions, and let 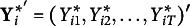 be the corresponding vector for the underlying variables. The latter is typically assumed to follow a multivariate normal distribution with mean vector 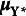 and covariance matrix 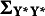 of size 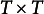 , such that the probabilities associated with the observed values of 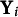 can be determined by the probability distribution of 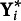 .

The auxiliary model establishes the connection between the observed ordinal response 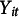 and the corresponding unobserved or latent continuous variable 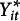 at each time point. This linkage is achieved through unknown cut-points or thresholds. Although 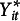 is assumed to follow a normal distribution, its mean and variance remain unidentified due to the limited availability of ordinal information.

For an ordinal variable 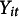 with *C* categories measured at *T* fixed occasions, there are 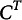 possible observed response patterns, not all of which may be observed in a given dataset. In this case, the sample data consists of the number of individuals with each of these response patterns. Any hypothesized model must explain (1) the univariate or marginal proportions, that is, the proportion of individuals in each of the *C* response categories for each of the *T* variables, and (b) the bivariate or joint proportions, that is, the proportions of individuals with each of the 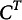 possible response pattern. The 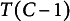 observed marginal proportions are insufficient for estimating the thresholds 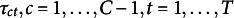 , or the parameters of the underlying variables, namely 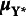 and diag( 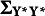 ), without imposing certain restrictions.

### 3.1 Identification issues

Constraints must be applied to achieve model identification, and this is where different parameterizations of the auxiliary model come into play. Various sets of conditions exist that are sufficient to ensure identification, and they vary based on assumptions made about the thresholds (whether they are time-varying, time-invariant, or fixed) and the means and variances of the underlying variables (whether they are fixed on all occasions, all free but one, or freely estimated on all time points). Different software may adopt distinct identification conditions, leading to variations in parameter estimates and model fit across programs. Table 2 details the main parameterizations in the literature that we discuss.

**Table 2:** Alternative sets of identification constraints for the auxiliary model in presence of ordinal data

Parameterization	Mean	St.dev.	Thresholds					No. of parameters
*Standard*	0	1	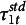	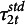	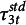	…	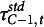	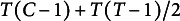
*Alternative 1*	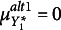	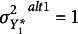	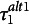	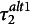	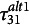	…	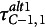	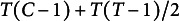
	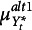	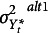	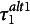	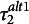	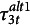	…	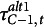	
*Alternative 1 - with thresholds invariance*	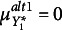 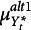	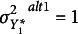 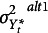	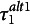	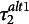	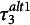	…	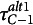	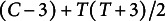
*Alternative 2*	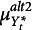	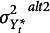	0	1	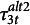	…	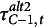	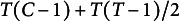
*Alternative 2 - with thresholds invariance*	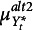	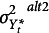	0	1	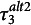	…	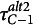	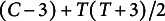

- ***Standard parameterization.*** The first row of Table 2 refers to the set of constraints commonly adopted in single-population or cross-sectional applications. It consists of fixing the expected value and standard deviation of 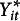 equal to zero and one, respectively, on each occasion. When this standard parameterization is adopted, the thresholds are freely estimated and assumed to be time-varying. These 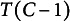 parameters are determined as percentiles of the standard normal distribution, whereas the 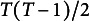 off-diagonal elements of 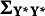 are estimated as polychoric correlations. This is the default parametrization applied in Mplus and lavaan, known as standard delta parameterization. Jöreskog (2001) also refers to it as standard parameterization, whereas Kamata and Bauer (2008) term it marginal parameterization since the marginal distribution of the continuous underlying variables is standardized.

One weakness of standard parameterization is that all underlying variables are standardized to have zero means and unit standard deviations. However, in the context of longitudinal data, where the response alternatives are the same across multiple time points, differences in the distribution of these variables can reflect differences in the means and/or variances of the underlying latent variables.

Researchers have proposed alternative identification constraints for categorical repeated measures, recognizing the limitations of the standard parameterization. These alternative parameterizations, which allow the means and variances of the underlying variables to vary freely, are a significant step toward a more accurate representation of the underlying data structure. Additional constraints are imposed, mainly on the thresholds of these variables, to ensure that the latent propensities are identified and comparable across time points. By imposing these constraints, analysts can establish a fixed reference point for the scale of the underlying response variables. This ensures that observed changes in the distributions reflect differences in the underlying variables, not inconsistencies in measurement.

- ***Alternative parameterization 1.*** The first alternative freely estimates the underlying variable’s means and variances on all occasions except on the first one (Millsap & Yun-Tein, 2004; Muthén & Asparouhov, 2002). That is, the underlying variable mean and variance at the first occasion, 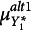 and 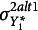 , are fixed to zero and one, respectively, while 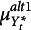 and 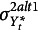 are estimated for 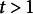 .^3^ These assumptions are sufficient to identify all the thresholds on the first occasion. On subsequent occasions, two thresholds are assumed to be time-invariant, *e.g.,* 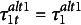 and 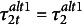 for all *t*. A notable feature is that complete invariance of all threshold parameters is not required. As discussed before, to estimate differences in the means and variances of latent variables over time—as done with latent growth models—one must ascertain that the underlying variables are on the same scale on different occasions. This is achieved by defining a common across-time metric in terms of the standard deviation on the first occasion.
  As shown in the second row of Table 2, the number of parameters to be estimated is the same as in the standard parameterization, with 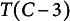 thresholds, 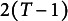 means and variances of the underlying variables, and 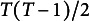 polychoric covariances, for a total of 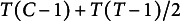 parameters. A one-to-one correspondence exists between the parameters in this alternative parameterization and those from the standard one. That is, on the first occasion, 
(2)

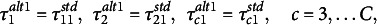

  whereas, on subsequent occasions, 
(3)

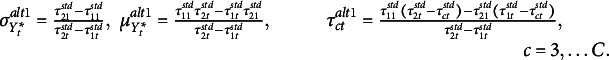

  Some authors claim that analysts should also estimate the thresholds but constrain the same threshold to be equal over time (Muthén & Muthén, 1998–2017, Example 6.5). That is, 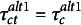 for 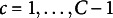 , and 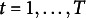 , and this parameterization is detailed in the third row of Table 2.^4^
  If the assumption of threshold invariance holds, then the alternative thresholds 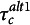 for each category, 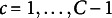 , should be equal to the corresponding threshold estimated on the first occasion under the standard parameterization ( 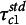 ), since the mean 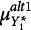 and standard deviation 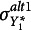 on the first occasion are set equal to zero and one, respectively. Based on this relationship, 

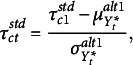

  or, equivalently, 
(4)

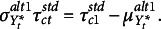

  Based on eq. (4), if thresholds are assumed to be equal over time, the ratio of the standard deviation of the underlying variable on two different occasions must be equal to the ratio of the differences between any two thresholds in the standard parameterization on these two occasions: 

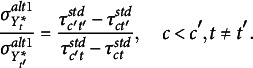

  In other words, if thresholds under the alternative 1 parameterization are truly invariant at times *t* and 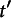 , the ratio of differences between any two standardized thresholds at the two time points should be equal. If any one of the thresholds for a time point is not invariant, then the equality mentioned above should not hold for the thresholds in the standard parameterization for that point. For the alternative 1 parameterization, for any pairs ( 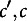 ) and ( 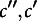 ), with 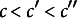 , we also obtain 
(5)

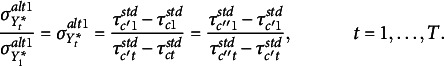

  Therefore, this equation can be considered as a test of the assumption regarding the lack of invariance of at least one of the thresholds for a given time point.
- ***Alternative parameterization 2.*** Jöreskog (2001) proposed an alternative specification for longitudinal data. It is based on defining the origin and unit of measurement of 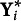 in terms of thresholds (Bollen & Curran, 2006; Fisher & Bollen, 2020; Mehta et al., 2004). The common practice is to fix the distance between the first two thresholds as one unit on the new scale, that is 
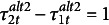
 , for all 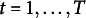 . Equivalently, the first threshold could be fixed to zero and the second at one on each occasion, indicating that 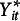 on this new scale must be greater than zero for an individual to be in an ordinal category greater than one. This allows us to recover, on each occasion, both the mean and variance of 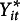 as well as the other ( 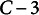 ) thresholds in this new measurement scale. Given a number of ordinal categories equal to or greater than three, these conditions are necessary and sufficient to ensure the identification of the model linking 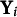 to 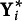 . This set of constraints also represents an alternative but equivalent parameterization of the standard one. Indeed, it can be easily shown that 
(6)

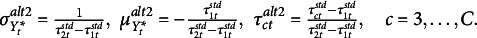

  A one-to-one relationship also stands between the two alternative parameterizations, as illustrated in Appendix A. In applying this parameterization in conjunction with a linear growth model for categorical data, Mehta et al. (2004) additionally imposed the assumption of threshold invariance over time. That is, 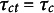 , for 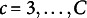 , and all *t*. As discussed by Jöreskog (2001) and Mehta et al. (2004), this assumption implies that 
(7)

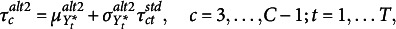

  where 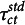 is the unconstrained threshold for the *c*th category at the *t* time point estimated under the standard parameterization. Eq. (7) sets constraints on 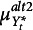 and 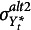 because the right-hand side varies with *t*, whereas the left-hand side does not. If 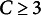 , the common thresholds, 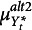 and 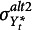 can be estimated from the univariate marginal data of those variables whose thresholds are supposed to be equal. Eq. (7) can be rewritten for any two thresholds *c* and 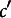 for any 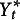 as 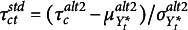 , and 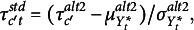 such that 

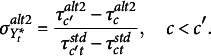

  It follows that the ratio of standard deviations on two different occasions is equal to the reciprocal of the ratio of the differences between any two standardized thresholds at those time points. For example, there are two independent equations with three thresholds at each time point. That is, for the pairs (2,1) and (3,2), we get 
(8)

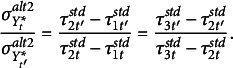

  If any one threshold for a time point under the alternative 2 parameterization is not invariant, eq. (8) fails to be true for that time point. Hence, this equation evaluates the lack of invariance of at least one of the thresholds at a given time point.

### 3.2 Binary case

Binary variables 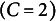 are special cases that require further comments. Indeed, with dichotomous variables, there are *T* available proportions, 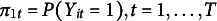 , to estimate the only threshold 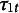 , the mean and variance of the underlying variables at each occasion.

When the standard parameterization is adopted, the only available threshold is freely estimated on each occasion, whereas the means and variances of the underlying variables are always set to zero and one, respectively. Hence, no different constraints are placed with respect to the categorical case (see the first row of Table 3).

**Table 3:** Alternative sets of identification constraints for the auxiliary model in presence of binary data

Parameterization	Mean	St.dev	Thresholds	No. of parameters
*Standard*	0	1	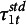	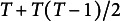
*Alternative 1 - Millsap and Yun-Tein (2004)*	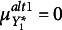	1	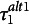	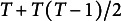
	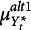			
*Alternative 1 - Muthén and Muthén (1998–2017)*	0	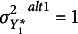	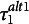	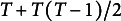
		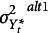		
*Alternative 2*	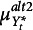	1	0	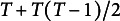

On the other hand, additional restrictions have to be placed for the alternative parameterizations. Millsap and Yun-Tein (2004) suggest freely estimating the means of the underlying variables on each occasion except on the first one, setting the variances of the underlying variables to one on each occasion and estimating the only available threshold under the assumption of time-invariance. These constraints are detailed in the second row of Table 3. Differently, Muthén and Muthén (1998–2017) suggest freely estimating the underlying variable variances on all the occasions except on the first one, where 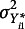 is fixed to one. They also suggest keeping the only available threshold invariant on each occasion. In this regard, the means of the underlying variables 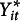 have to be fixed to zero for the auxiliary model to be identified (see the third row in Table 3).

In the presence of binary data, it is impossible to implement the threshold constraints proposed by Jöreskog (2001), being only one threshold available per occasion. Even when holding thresholds invariant over time, this does not identify both means and variances on all occasions. Jöreskog (2001) proposes to fix the thresholds equal to zero and the variances 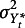 to one on all occasions, only allowing the means 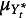 to vary over time. This is detailed in the last row in Table 3.

### 3.3 Estimation

SEMs for continuous endogenous variables analyze the mean vector 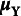 and covariance matrix 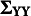 of the observed indicators. However, when one or more of the endogenous observed variables are categorical, the analysis shifts to the mean vector 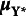 and covariance matrix 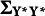 corresponding to 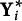 . If consistent estimators for 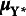 and 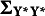 are available, researchers can analyze them similarly to continuous indicators. Consequently, the estimation procedure comprises two distinct steps. The first step obtains consistent estimates of the means 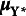 and covariance matrix 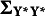 for 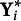 . To perform significance testing, analysts also need the asymptotic covariance matrix of the elements in 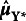 and 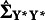 . Once the means, variances, and covariances of 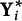 are in hand, researchers can estimate the parameters of any longitudinal model they apply to 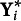 .

Here, we focus on the first step needed to estimate 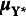 and the unconstrained covariance matrix 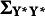 . As we discussed in Section 3.1, distributional assumptions and identification constraints are necessary for estimation. Assuming a bivariate standard normal distribution for each pair of variables in 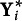 facilitates the estimation of thresholds and polychoric correlations/covariances for noncontinuous variables. Univariate standard normality for each underlying variable 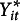 enables threshold estimation as percentiles of the standard normal distribution: 

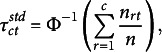

where 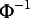 is the quantile function of the standard normal distribution, 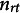 is the number of cases in the *r*th category at time *t*, and *n* is the sample size. While univariate margins are valuable for estimating the thresholds in the standard parameterization, bivariate tables are essential for estimating the correlation between 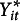 and 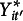 . Following Olsson (1979), the log-likelihood for the polychoric correlation is given by 

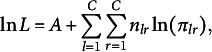

where *C* is the number of categories for both 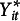 and 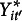 , 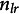 is the number of cases in the 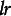 th cell of the bivariate table, and *A* is an irrelevant constant that does not influence the values that maximize the likelihood. Hold the thresholds fixed at the estimates from the univariate margins, 

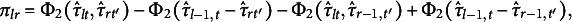

where 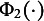 is the bivariate normal distribution function with correlation 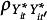 . As a result of this conditional estimation procedure, 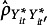 , for 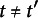 , is a pseudo-maximum likelihood estimate.

When the means and variances of each 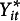 are of interest, it is possible to estimate these quantities rather than constraining them to 0 and 1, respectively. The alternative parameterizations of the auxiliary model allow this option. In the standard parameterization, assuming 
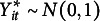
 at each occasion allows for estimation for the mean (keeping the variance 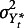 fixed to one) by shifting the distribution of 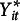 by a constant 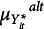 . This results in the transformed distribution as 
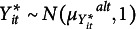
 . If, in addition to the mean, the variance of 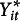 is estimated, the distribution of 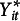 is shifted and scaled, such that 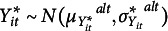 . Hence, in the alternative parameterization of the auxiliary model, the corresponding thresholds, means, and variances of the underlying variables are based on their relationships with the thresholds of the standard parameterization. These relationships are given in Section 3.1 and detailed in eqs. (2) and (3) for the alternative parameterization proposed by Millsap and Yun-Tein (2004), and in eq. (6) for the parameterization proposed by Jöreskog (2001). If thresholds are assumed to be invariant over time, additional constraints are placed on the means and variances of the underlying variables under the alternative parameterizations, as discussed in Section 3.1.

The polychoric covariance matrix of the underlying variables under the alternative parameterization is then determined by scaling the polychoric correlation matrix 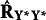 estimated for the standard parameterization as follows: 

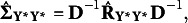

where 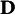 is a 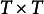 diagonal matrix with generic element 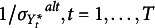 .

### 3.4 Illustrative example: auxiliary model for NLSY97 data

Returning to our motivating NLSY97 example, we estimate the auxiliary measurement model connecting the binary and ordinal variables to their underlying continuous variables. The underlying variables 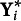 represent propensities such as the inclination to use illegal drugs, experience depression, or perceive good/excellent health status. In the case of the illegal drug use variable, the threshold is the point that separates use from nonuse of illegal drugs. An individual whose propensity exceeds the threshold uses illegal drugs, whereas those who fall below it do not. Similarly, for the other two categorical variables, when the latent propensity or perception falls between thresholds 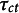 and 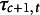 on a given occasion, the observed ordinal response corresponds to category *c*.

As discussed in Section 3.1, various parameterizations of the auxiliary model are available. Both the standard auxiliary model and alternative parameterizations, where no threshold invariance constraints are imposed, result in just identified models (with zero degrees of freedom) that perfectly fit the data. When researchers fit these to the data, they obtain estimates of unknown thresholds, means of underlying variables, their variances, and polychoric correlations/covariances. For illustrative purposes, we report—in the Supplementary Material, due to space constraints—the estimated means and polychoric correlations/covariances for the auxiliary model that jointly considers all three observed variables based on the standard and alternative parameterizations. For the latter, we consider the models with and without threshold invariance.

The assumption of threshold invariance is tested by estimating the auxiliary model in which the same threshold at all time points is restricted to be equal. It is worth noting that both alternative (1 or 2) parameterizations of the auxiliary model are equivalent in their chi-square and degrees of freedom. Table 4 provides fit statistics for each categorical variable’s auxiliary model based on the alternative (1 or 2) parameterization with threshold invariance. For the binary variable, threshold invariance is mandatory for the auxiliary model to be just identified and is not testable. Under the assumption of threshold invariance, we have also estimated the multivariate auxiliary model for the three variables, which includes the estimation of thresholds, means, and variances for each variable, along with the polychoric covariances among all the underlying variables. Note that the threshold invariance hypothesis is not rejected for depressive symptoms at any conventional level and that for general health status is marginally significant. With over 5000 cases, statistical power should be high. We also checked the fit indexes and found that the CFI, TLI, and RMSEA all suggest excellent fit. The negative value of the BIC ( 
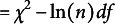
 ) also support the invariant threshold models (Raftery, 1995).

**Table 4:** Fit statistics for the alternative(1 and 2) auxiliary models based on threshold invariance estimated for depressive symptoms, general health status, and for all the three variables jointly considered

Fit statistics	Depressive symptoms	General health status	Joint auxiliary model
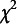	6.213	19.450	25.849
df	5	10	15
*p*-value	0.286	0.035	0.040
CFI-TLI	1.000–0.997	1.000–0.999	1.000–0.997
RMSEA	0.007	0.013	0.012
BIC	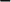 36.673	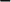 66.322	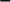 102.808

These results suggest that the auxiliary model under alternative 1 or 2 with invariant thresholds is the most promising structure to use for these data when moving to the longitudinal model. However, these results are only with regard to the auxiliary model. In the following Sections, we consider the possible identification constraints of the different longitudinal models, and we turn to this topic next.

## 4 A general longitudinal model for categorical repeated measures

Given the latent response variates 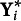 , we can apply the ALT model (Bollen & Curran, 2004). It encompasses the classical autoregressive and latent growth models as special cases. Following the general representation provided by the authors, the unconditional model is specified through two equations 
(9)

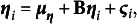



(10)

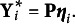

In eq. (9), 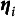 is a vector that includes both the underlying variables 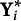 and the random growth components 
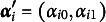
 . 
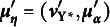
 represents a vector of intercepts and means, and the 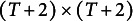 matrix 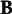 specifies the coefficients for the relationships between the elements of 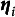 . It is divided into sub-matrices as follows 

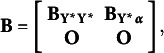

where 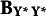 contains the autoregressive effects among the underlying variables 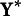 , that is 

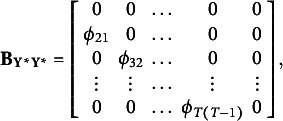

and 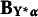 relates the underlying variables 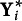 to the random growth components.

The autoregressive relations in the ALT model give rise to an initial condition problem since the variable at the start of the observation period, 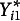 , should be affected by the random intercept and slope as well as unavailable pre-sample latent responses, say 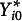 . To handle this problem, we can assume 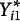 to be predetermined, correlated with the growth components, or treated as endogenous. We consider 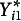 predetermined, knowing that, using rules from Lee and Hershberger (1990) and Hershberger (2006), the unconditional autoregressive latent trajectory model with 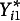 predetermined or 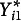 endogenous are (globally and covariance) equivalent (Bianconcini & Bollen, 2018). Hence, considering a *linear* growth curve, 
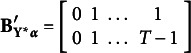
 .

The disturbance vector for 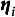 is 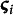 , that is 
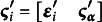
 . It is assumed to have a zero mean vector, and its covariances depend on the model. Under the assumption that 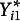 is predetermined, 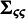 is a block-diagonal matrix of the form 
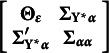
 , where 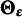 is a diagonal matrix with error variances that can differ over time, such that 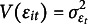 , for 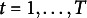 , 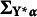 contains the covariances between the predetermined 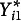 and the random components 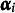 , such that 
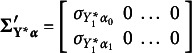
 , and 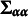 is the full and symmetric covariance matrix of the growth components.

Equation (10) links the underlying response variates 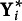 to the latent variables in 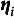 through the matrix 
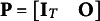
 , where 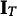 is an identity matrix with dimensions that depend on the number of repeated measures and 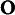 is a zero matrix of dimensions 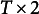 . These assumptions lead to the following implied moments for the underlying variables in the autoregressive latent trajectory model of 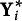



(11)

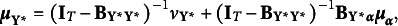



(12)

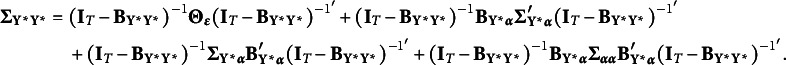

The first term on the right-hand side of eqs. (11) and (12) are the moments implied by the autoregressive of order one component of the model, whereas the other terms account for the interaction between the autoregressive and growth components. The implied moments help determine the identification of the model parameters, test the model fit, and prove if the choice of different parameterizations is arbitrary or not.

*Multivariate autoregressive latent trajectory model.* If two or more binary or ordinal repeated measures are observed, the ALT model has to be generalized to deal with multiple series. To illustrate and clarify this model specification, consider that we have two series of repeated ordinal variables and that their underlying variables have autoregressive and cross-lagged relations with each other. Say that 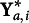 contains the series of longitudinal underlying variables for the first series and 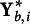 contains the longitudinal underlying variables for the second one. Based on our empirical application, we can consider 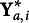 to be the underlying variables associated with the observed depressive symptoms, while 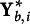 relates to the observed general health status variables. Let 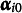 and 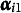 be vectors of the growth components for the two series. In this situation, we write 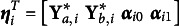 , and the vector 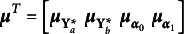 , where the 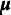 ’s are intercepts of endogenous variables or means of latent variables, and the subscript signifies which variable. Next is the 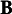 matrix, 

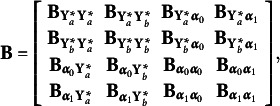

which is partitioned. As before, the first subscript of the coefficients shows the variables receiving effects, while the second subscript is the variables emitting effects. For instance, 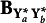 gives the coefficients of the direct effects of the second series of repeated variables on the first series, this would include any cross-lagged effects from **Y**_*b*, i_^*^ to **Y**_*a*, i_^*^. Similarly, 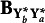 gives the direct effects of the first series on the second and 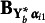 contains the coefficients of the direct effects of the random slopes of both series on the repeated measures of the second series. Knowing that none of the repeated measures has direct effects on the random intercepts or random slopes, and no direct effects are estimated for the random components, all coefficients that correspond to such effects are set to zero; that is, 

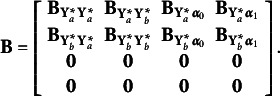

The vector 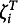 is now given by 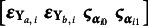 . The first two elements, 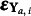 and 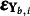 , are disturbances or errors of the two repeated measures series. These errors are correlated when referring to the same occasion (*concomitant effects*). The last two vectors, 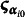 and 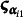 , are the disturbances or errors from the equations for 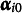 and 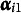 , assumed to be all correlated. In brief, the preceding equations enable the analysis of two repeated measures with binary or ordinal variables. Generalizing the model to analyze three or more series of repeated measures is straightforward.

### 4.1 Identification issues

The ALT model for continuous measures requires constraints to ensure its identification, particularly with fewer than five waves of data (Bollen & Curran, 2004). Extending this model—and its special cases—to ordered categorical measures presents additional challenges. We can approach the identification problem by dividing the constraints needed into two parts. The first part contains constraints to identify 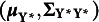 . The second part contains conditions to identify the autoregressive latent trajectory model parameters. For the latter, we will also discuss the identification issues for the linear latent growth and autoregressive of order one models.

Once the auxiliary model is accurately identified and estimated, we treat the means and covariances between the underlying variables 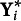 as known and use them to identify the longitudinal model for 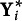 .

This section outlines identification conditions for the linear latent growth, the first-order autoregressive, and ALT models. We explore alternative parameterizations for these models based on the previously discussed auxiliary model specifications. Our assessment focuses on whether distinct specifications lead to equivalent models. In SEMs, equivalent models generate identical model-implied moment matrices and equally fit the data, with equal test statistics, fit indexes, and degrees of freedom (Lee & Hershberger, 1990; Stelzl, 1986). Equivalent models impose the same constraints on the population covariance matrix, known as 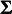 constraints (Steiger, 2002). Models with the same 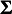 constraints are 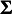 -equivalent since they cannot be empirically distinguished.

A formal definition of model equivalence has been provided by Raykov and Penev (1999), who established a necessary and sufficient condition based on the existence of a parameter transformation that preserves the implied covariance matrix and covers the entire parameter space of the other model. This implies that equivalent models remain invariant under this transformation. Validating this condition involves deriving implied covariance matrices, equating corresponding elements, and solving for parameters to confirm the 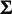 -equivalence. If there is no mapping, the two models are not equivalent. An extension of the rule by Raykov and Penev (1999) for identifying equivalent models with a mean structure has been discussed by Levy and Hancock (2007) and Losardo (2009) with a specific focus on the equivalence of latent curve model specifications. We follow the approach of Losardo (2009), accommodating the presence of categorical repeated measures.

#### 4.1.1 Linear growth model

We begin by considering the linear latent growth model for 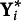 . Three different specifications correspond to the standard and alternative parameterizations of the auxiliary model. Despite the time-specific scale of thresholds in the standard parameterization, we consider it being the default option in Mplus 8.6 and lavaan 0.6-16.

To derive identification constraints for each alternative specification, as detailed in Table 5, we need the mean and covariance matrix implied by the linear growth model. These are given by 
(13)

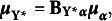



(14)

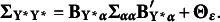

**Table 5:** Identification constraints and degrees of freedom for the linear growth model for categorical repeated measures

Constraints Auxiliary model	Standard		Alternative 1		Alternative 2	
			(Threshold invariance)		(Threshold invariance)	
Stage 1	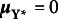		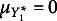		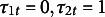	
	diag( 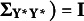		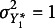		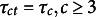	
			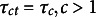			
Stage 2	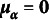		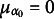			
No. of parameters	T(C-1)+3		C+T+2		C+T+2	
Degrees of freedom	[T(T-1)/2]-3		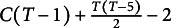		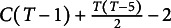	

For a deeper understanding of the relationship between these specifications, Table 5 also reports the corresponding degrees of freedom.

The WLSMV estimator only uses information coming from the first- and second-order sample statistics corresponding to the 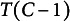 univariate proportions and the 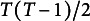 polychoric correlations. The degrees of freedom of a given specification are the difference in the number of available information, 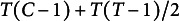 , and the number of parameters in the considered model. It is important to note that not all three specifications have the same degrees of freedom. Hence, the standard parameterization would not provide an equivalent specification to the alternative ones. The assumptions made for the identification of the auxiliary model impact the specification and interpretation of the linear latent growth model defined for 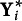 . The two alternative parameterizations provide equivalent specifications to each other since a one-to-one relationship between their parameters exists, as detailed in Table 6.

**Table 6:** Parameter transformations for the linear growth models based on the parameterization proposed by Muthén and Muthén (1998–2017) (Model A) and the one illustrated by Mehta et al. (2004) (Model B)

*Model A in terms of Model B*	*Model B in terms of Model A*
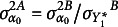	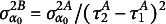
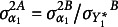	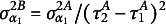
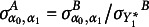	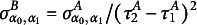
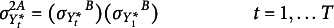	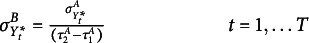
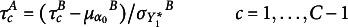	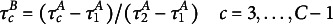
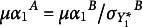	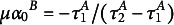
	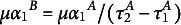

When adopting the standard parametrization (second column of Table 5), Stage 1 constraints are sufficient to identify the auxiliary model. Setting ( 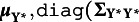 )) to ( 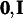 ) identifies the thresholds 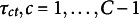 , on all occasions. Stage 2 constraints involve fixing 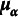 to 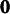 to satisfy the implied mean condition in eq. (13). Appendix B shows that these conditions are sufficient to identify the linear latent growth model., followed by the fact that since the implied mean is a function of the constant matrix 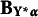 and of the vector of growth means 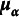 , the latter has to be fixed equal to zero for the implied mean condition in eq. (13) to be satisfied.

A key distinction between SEMs for continuous outcomes and models for categorical data lies in the identification of the error 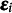 variances, 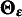 . The variances of the errors cannot be independently identified from the variances of the underlying variables, diag( 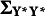 ), as 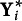 is a latent vector without an inherent scale. If diag( 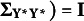 , the variance–covariance matrix of the growth components 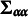 is correctly identified, while the error variances 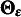 are determined as a remainder based on eq. (14).

An alternative set of identification constraints replaces diag( 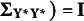 with 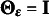 . It is denoted as (standard) theta parameterization in Mplus 8.6 and lavaan 0.6-16 and termed conditional parameterization by Kamata and Bauer (2008). This approach assumes standardized conditional distributions of 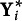 given 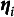 , with marginal variances of 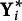 obtained as the remainder based on eq. (14). In longitudinal data, this assumption could be more suitable in that the variances of 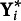 are permitted to vary over time while the error variances are not, and this latter assumption might be more plausible. Of course, if there are reasons to think that these error variances differ over time, this assumption is also not desirable.

To address the conflicting conclusions drawn by Grimm and Liu (2016), Lee et al. (2018) and Newsom and Smith (2020) regarding the relationship between these standard parameterizations of the linear growth model, we provide a theoretical illustration. We limit our analyses to models with four time points to avoid complex and unproductive mathematical details. We compare the two linear growth model parameterizations (based on diag 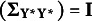 or 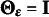 ) in terms of 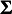 and 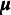 constraints. Following Steiger (2002), the covariance matrix and the mean vector implied by each standard (delta and theta) model specification are first derived and reported in the Supplementary Material. Both model specifications have no 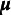 constraints and 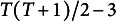 (seven) 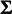 constraints corresponding to the degrees of freedom on the covariance structure of the model, as illustrated in Table 7.

**Table 7:** 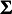
 *constraints for the linear growth model based on the (standard) delta parameterization* (diag( 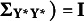 ) *and the (standard) theta parameterization* ( 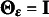 )

*Variances*	diag( 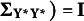	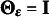
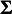 constraints		
1	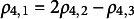	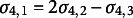
2	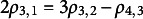	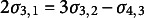
3	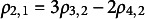	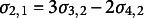
4	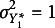	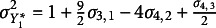
5	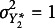	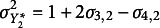
6	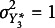	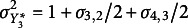
7	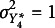	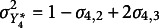

It is evident that these two standard specifications of the linear growth model are not empirically equivalent, as the constraints on the underlying variable variances are different, and the 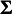 constraints derived when all the underlying variable variances are fixed to one are defined in terms of polychoric correlations 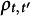 .

When adopting the alternative parameterization proposed by Muthén and Asparouhov (2002) and by Millsap and Yun-Tein (2004) for the auxiliary model, which fixes the mean of the underlying variable to zero only on the first occasion, the implied mean condition in eq. (13) necessitates constraining only the mean of the intercept component, 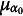 , to zero (refer to the third column of Table 5). The variance of the underlying variable on the first occasion is set to one, while the other ( 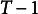 ) variances are freely estimated. Due to the interdependence of 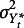 and 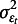 , an alternative specification is obtained by placing these variance restrictions on the errors rather than on the underlying variable. In Appendix B, we prove that these conditions are sufficient for the model identification.

In contrast to the standard parameterization, it can be demonstrated that placing restrictions on diag( 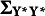 ) or 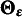 defines two specifications of the linear growth model that are equivalent. Table 8 presents the parameter transformations for the model where all underlying variable variances but one are freely estimated (Model C) and the model where all error variances are free parameters except on the first occasion (Model D).

**Table 8:** Parameter transformations for the linear growth models where all underlying variable variances are free except on the first occasion (Model C) and when all error variances are free except at the first time point (Model D)

*Model C in terms of Model D*	*Model D in terms of Model C*
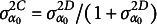	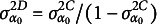
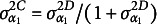	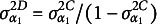
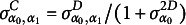	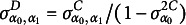
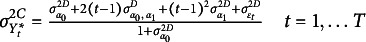	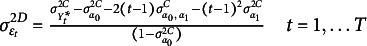
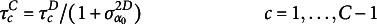	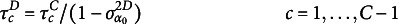
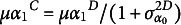	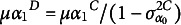

While these transformations are not identity functions, it is evident that parameters in Model C are functions of the corresponding parameters in Model D divided by the underlying variable variance on the first occasion under that specification, that is, 
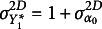
 . Conversely, parameters in the theta specification are functions of the corresponding parameters in the Model C parameterization divided by 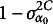 , where 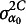 represents the intercept variance in the model where all underlying variable variances are freely estimated except on the first occasion.

Finally, following the alternative 2 parameterization suggested by Jöreskog (2001), where all means and variances of the underlying variables are freely estimated, no restrictions are needed for the identification of the growth parameters, as detailed in the fourth column on Table 5 and proven in Appendix B. For this specification, focusing on either the 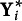 or error variances yields the same 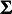 and 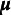 constraints, as illustrated in Table 9. Hence, these two alternative linear growth model specifications are empirically equivalent, with parameters of the two specifications related via identity transformations.

**Table 9:** 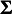
 *and* 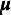 *constraints for the linear growth model based on the alternative 2 delta parameterization* (diag 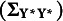 *freely estimated) and the alternative 2 theta parameterization* 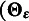 *freely estimated)*

*Variances*	diag( 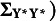 free	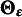 free
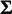 constraints		
1	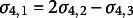	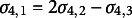
2	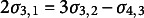	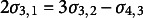
3	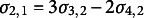	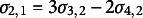
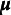 constraints		
1	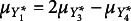	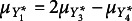
2	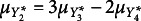	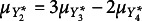

*Binary data*. A special case that requires further comments arises when the measured variables are dichotomous 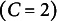 . Four different specifications of the linear latent growth model can be derived based on the various parameterizations of the auxiliary model detailed in Table 3. Identification constraints for these specifications are provided in Table 10, along with the corresponding degrees of freedom. The degrees of freedom are computed as the difference between the number of available information, equal to 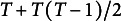 , and the number of parameters in each model.

**Table 10:** Identification constraints and degrees of freedom for the linear growth model for binary data

Constraints Auxiliary model	Standard		Alternative 1		Alternative 1		Alternative 2	
			Millsap and Yun-Tein		Muthén and Muthén			
			(2004)		(1998–2017)			
Stage 1	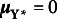		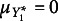		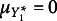			
	diag( 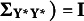		diag( 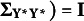		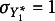		diag( 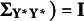	
			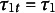		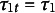		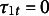	
Stage 2	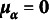		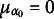		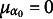			
No. of parameters	T+3		5		T+4		5	
Degrees of freedom	[T(T-1)/2]-3		[T(T+1)/2]-5		[T(T-1)/2]-4		[T(T+1)/2]-5	

All the constraints in Table 10 are sufficient for identifying the corresponding linear latent growth model. The proof follows the same line as detailed in Appendix B in the presence of categorical data. Attention should be paid to the alternative specification suggested by Muthén and Muthén (1998–2017), whose constraints are reported in the fourth column in Table 10. Due to the dependence of the underlying variable moments 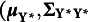 ) on the parameters of the growth model 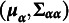 , based on eqs. (13) and (14), we can employ fewer Stage 1 constraints than those outlined in Table 10 by taking advantage of the model structure. In this case, the identification problem is treated simultaneously for the thresholds, 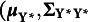 ), and for the latent growth parameters 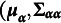 ). A formal proof is provided in Appendix B.

It is important to note that only the alternative parameterization proposed by Millsap and Yun-Tein (2004) and the one suggested by Jöreskog (2001) share the same number of degrees of freedom. Furthermore, they are equivalent specifications of the linear growth model, since a surjective transformation exists that expresses the parameters of one specification as a function of those of the other and vice versa. All the parameters ( 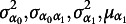 ) are related by identity functions except for the threshold 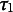 in the alternative parameterization by Millsap and Yun-Tein (2004) that is equal to minus the intercept mean 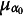 in the Jöreskog (2001) parameterization and vice versa.

For each parameterization, an alternative set of sufficient conditions can be derived by replacing the constraints on diag( 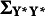 ) with corresponding constraints on the error variances 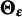 . The results derived for categorical data directly apply when the models are fitted to binary observations: anytime the underlying variable or error variances are all fixed to one, the two alternative parameterizations of the linear growth model are not empirically equivalent. This occurs when the standard parameterization, the alternative one proposed by Millsap and Yun-Tein (2004), and that suggested by Jöreskog (2001) are considered. On the other hand, for the parameterization suggested by Muthén and Muthén (1998–2017), the two parameterizations based on freely estimating all but the first underlying variable or error variances are empirically equivalent. The parameters of the two specifications can be expressed as one function of the other, as detailed in Table 8.

#### 4.1.2 First-order autoregressive model

We now consider alternative parameterizations for the first-order autoregressive model. In situations where the dependent variable is influenced by or influences other dependent variables, assumptions need to be placed on the error variances due to the improper parameter constraints that come into play when focusing on the variances of underlying variables (Muthén and Muthén, 1998–2017, pp. 485–486). Specifically, when autoregressive components are present, Mplus exclusively supports theta parameterizations.

The mean and covariance matrix implied by the first-order autoregressive model can be expressed as follows 
(15)

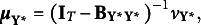



(16)

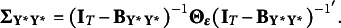

Here, 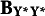 contains all autoregressive effects among underlying variables, such that 
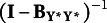
 has the following specific structure 

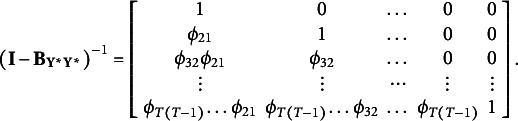



Identification conditions for different autoregressive model specifications, along with corresponding degrees of freedom, are presented in Table 11. Appendix B proves the sufficiency of these constraints for model identification. Degrees of freedom are computed based on the difference between information from the first- and second-order statistics, equal to 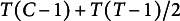 , and the number of parameters in each specification.

**Table 11:** Identification constraints and degrees of freedom for the autoregressive model for categorical repeated measures

Constraints Auxiliary model	Standard		Alternative 1		Alternative 2	
			(Threshold invariance)		(Threshold invariance)	
Stage 1	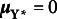		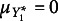		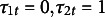	
			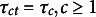		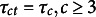	
Stage 2	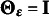		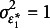			
	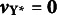		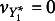			
No. of parameters	T(C-1)+(T-1)		C+3T-4		C+3T-4	
Degrees of freedom	(T-2)(T-1)/2		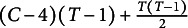		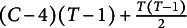	

Coherently with the analysis performed for the linear latent growth model, we still consider alternative parameterizations based on the assumption of threshold invariance. Understanding how constraints on the auxiliary model imply different or equivalent autoregressive model specifications is crucial. Alternative parameterizations endow the model with 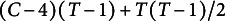 degrees of freedom, while the standard (theta) parameterization, which involves fixing the error means and variances on all occasions, has 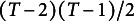 degrees of freedom.

When the assumption of threshold invariance is relaxed, a notable distinction is observed between the linear latent growth and autoregressive models. The standard and alternative autoregressive specifications are equivalent, characterized by 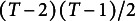 degrees of freedom. This is unique to the autoregressive model, as the standard specification of the linear growth model for categorical data still differs from the alternative ones.

In the Supplementary Material, we illustrate the model-implied mean and covariance structures for each autoregressive model, focusing on a simplified scenario with four observed time points. Based on these implied moments, we show that the alternative 1 (Model E) and 2 (Model F) parameterizations, both based on threshold invariance, share the same 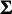 constraints, represented by 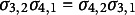 , 
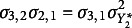
 , and 
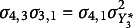
 . Table 12 outlines the relationships between parameters of these equivalent autoregressive model specifications.

**Table 12:** Parameter transformations for the autoregressive models based on the parameterization proposed by Muthén and Muthén (1998–2017) (Model E) and the one illustrated by Mehta et al. (2004) (Model F)

*Model E in terms of Model F*	*Model F in terms of Model E*
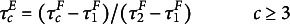	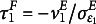
	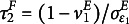
	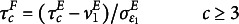
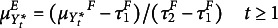	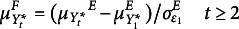
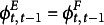	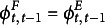
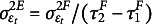	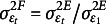

It is evident that Model F parameters are functions of the estimated error variance on the first occasion in Model E, while Model E parameters depend on the distance between the two first thresholds in Model F.

*Binary data.* The distinct characteristics of various parameterizations of the autoregressive model of order one become more apparent when applied to binary data. In this context, all considered specifications yield the same degrees of freedom, as presented in Table 13. It is straightforward to demonstrate that the parameters of each model can be expressed as functions of the others. To simplify the presentation, Table 14 just illustrates how parameters in the standard parameterization (Model G) relate to those in each of the alternative parameterizations.

**Table 13:** Identification constraints and degrees of freedom for the autoregressive model for binary data

Constraints Auxiliary model	Standard		Alternative 1		Alternative 1		Alternative 2	
			Millsap and Yun-Tein		Muthén and Muthén			
			(2004)		(1998–2017)			
Stage 1	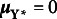		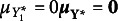		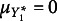		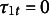	
			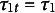		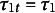			
Stage 2	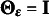		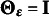		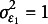		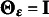	
	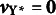		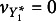		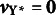			
No. of parameters	2T-1		2T-1		2T-1		2T-1	
Degrees of freedom	[T(T-3)/2]-1		[T(T-3)/2]-1		[T(T-3)/2]-1		[T(T-3)/2]-1

**Table 14:** Parameter transformations for the autoregressive model of order one for binary data based on the standard parameterization of the auxiliary model (Model G), following the alternative parameterization proposed by Millsap and Yun-Tein (2004) (Model H), that proposed by Muthén and Muthén (1998–2017) (Model I), and the one proposed by Jöreskog (2001) (Model L)

*Model G in terms of Model H*	*Model H in terms of Model G*
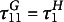	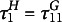
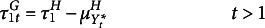	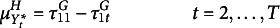
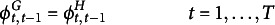	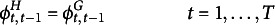
*Model G in terms of Model I*	*Model I in terms of Model G*
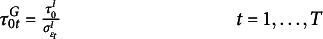	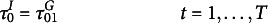
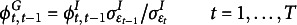	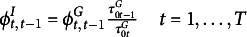
	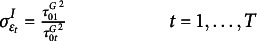
*Model G in terms of Model L*	*Model L in terms of Model G*
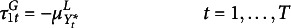	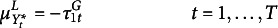
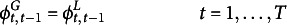	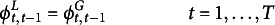

#### Autoregressive latent trajectory model

The ALT model integrates components from the previously discussed models, the linear latent growth and the first-order autoregressive model. Including autoregressive effects requires constraints to be specifically placed on the error variances rather than on the underlying variable variances, as discussed earlier.

In eqs. (11) and (12), the mean vector and covariance matrix implied by the ALT model are directly influenced by the autoregressive component. This influence is evident through terms such as 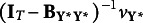 and 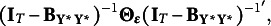 respectively.

Additionally, both moments depend on the interaction between the autoregressive and growth components. The Supplementary Material provides a detailed presentation of these moments for various ALT model specifications under the simplified scenario of a stationary autoregressive process with four observed occasions.

Table 15 presents identification conditions for different ALT parameterizations. These conditions are sufficient for identification. They can be easily proven in a manner similar to that for the linear latent growth and autoregressive model in Appendix B. The degrees of freedom for each specification are also reported. Consistent with the previous analysis, we consider alternative parameterizations based on the assumption of threshold invariance.

**Table 15:** Identification constraints and degrees of freedom for the autoregressive latent trajectory model for categorical repeated measures

Constraints Auxiliary model	Standard		Alternative 1		Alternative 2	
			(Threshold invariance)		(Threshold invariance)	
Stage 1	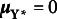		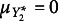		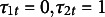	
			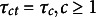		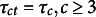	
Stage 2	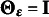		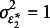			
	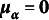		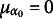			
	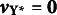		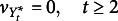		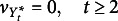	
No. of parameters	TC+4		C+2T+4		C+2T+4	
Degrees of freedom	[T(T-3)/2]-4		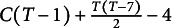		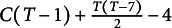	

As observed for the linear growth model, both alternative parameterizations share the same degrees of freedom, equal to 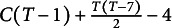 , while the standard parameterization has 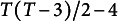 degrees of freedom. The model requires five occasions for identification without imposing additional parameter constraints beyond those outlined in Table 15.

A notable difference from both the linear latent growth and autoregressive model is that the two alternative parameterizations of the ALT model are not empirically equivalent. They exhibit distinct 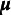 and 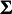 constraints. To illustrate this point clearly, we consider the simplified case of a stationary first-order autoregressive model with five observed occasions. The 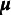 constraints for the different autoregressive latent trajectory specifications are illustrated in the following Table 16.

**Table 16:** 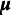
 *constraints for the different autoregressive latent trajectory model specifications*

*Parameterization*	Standard	Alternative 1	Alternative 2
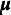 constraints			
1	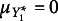	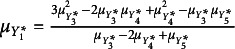	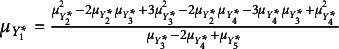
			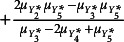
2	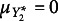	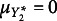	
3	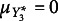		
4	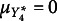		

The three ALT specifications also exhibit different 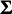 constraints, which are detailed in the Supplementary Material due to space constraints. Attempting to establish a correspondence between the parameters of the two alternative parameterizations, as requested by Raykov and Penev (1999), proves unsuccessful when equating corresponding elements in the implied covariances matrices. Consequently, the assumptions made for the identification of the auxiliary model strongly influence the specification and interpretation of the ALT model for 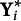 .

*Binary data*. Moving on to the analysis of binary data, the ALT model inherits the discrepancies observed in the alternative specifications for categorical data. In this context, none of the proposed parameterizations of the auxiliary model leads to equivalent specifications of the ALT model. Despite the fact that the alternative specifications proposed by Millsap and Yun-Tein (2004) and Jöreskog (2001) yield the same degrees of freedom, as presented in Table 17, the parameters of each model cannot be expressed as functions of the others, as required by Raykov and Penev (1999).

**Table 17:** Identification constraints and degrees of freedom for the autoregressive latent trajectory model for binary data

Constraints Auxiliary model	Standard		Alternative 1		Alternative 1		Alternative 2	
			Millsap and Yun-Tein		Muthén and Muthén			
			(2004)		(1998–2017)			
Stage 1	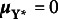		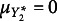		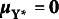			
			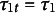		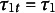		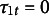	
Stage 2	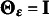		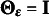		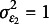		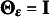	
	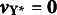		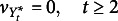		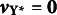		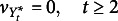	
	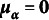		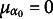		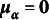			
No. of parameters	2T+4		T+7		2T+4		T+7	
Degrees of freedom	[T(T-3)/2]-4		[T(T-1)/2]-7		[T(T-3)/2]-4		[T(T-1)/2]-7	

Table 17 provides sufficient identification conditions for each model, along with corresponding degrees of freedom. Five waves of data are necessary—under each parameterization—to identify the model without placing additional constraints on the model parameters. This is coherent with what was observed in the presence of continuous observations (Bollen & Curran, 2004). Based on these findings, researchers should carefully consider the implications of selecting specific parameterizations of the autoregressive latent trajectory model, as evidenced by the NLSY97 data analysis, where different choices significantly influenced the interpretation of the results.

### 4.2 Estimation

The correlation matrix (standard parameterization) or the mean vector and unconstrained covariance matrix (alternative parameterizations) estimated based on the auxiliary model specification—as described in Section 3.3—are utilized to derive point estimates for the parameters of the dynamic model specified for 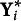 . Various estimators apply to estimate the structural parameters in a SEM. We specifically focus on the Diagonally Weighted Least Squares (DWLS) estimator, which is the default choice in Mplus 8.6 and lavaan 0.6-16. Once 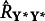 or ( 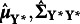 ) are available, all parameters of SEM are estimated simultaneously.

To begin, we organize the estimated means 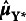 and all the diagonal and below diagonal elements in 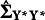 (or 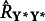 ) into a vector 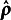 . Similarly, we place the implied moments 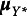 and 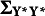 in a vector 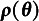 , where 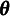 contains the SEM parameters. The DWLS estimator is then determined by minimizing 

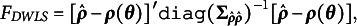

where 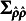 is the asymptotic covariance matrix of 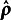 whose derivation has been widely detailed in Muthén (1984), Jöreskog (1994), and Muthén and Satorra (1995).

The parameters 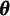 are chosen to minimize the weighted sum of squared deviations of 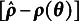 . The 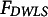 is consistent, asymptotically unbiased, and normally distributed (Browne, 1984). However, it lacks asymptotic efficiency. Default standard errors are no longer accurate, and goodness of fit tests no longer follow a 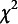 distribution. To address this, robust standard errors are obtained by considering the following sandwich-type asymptotic covariance matrix of the parameter estimates 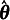 (Muthén et al., 1997) 

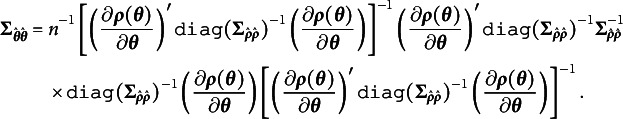

The square root of the main diagonal at the estimated parameters represents the robust standard errors of the parameter estimates. Mean and variance-adjusted chi-square statistics have been proposed to approximate the shape of the test statistics to the reference chi-square distribution with the associated degrees of freedom. The WLSMV estimator, which is the default estimator in Mplus 8.6 and lavaan 0.6-16 for models with endogenous categorical variables, relies on the Satterthwaite (1941) type correction. We refer the reader to Satorra and Bentler (1994) and Muthén et al. (1997) for its detailed description.

## 5 Illustrative example: longitudinal model parameterizations

To substantiate the theoretical insights presented in the paper, we applied the multivariate ALT models using both the standard and alternative (1 and 2) parameterizations, where threshold invariance is assumed. The results for illegal drug use (*drug*), depressive symptoms (*depr*), and general health status (*health*) are presented in Table 18. The different fit statistics for these models highlight their distinct specifications.

**Table 18:** *(Significant) parameter estimates for different parameterizations of the ALT model.*[ 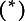 : *significant at 5% level.* 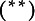 : *significant at 1% levelNote*: 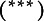 : *significant at 0.1% level.*]

	Standard		Alternative 1		Alternative 2			Standard		Alternative 1		Alternative 2	
*Parameters*			Threshold invariance		Threshold invariance		*Parameters*			Threshold invariance		Threshold invariance	
**C**ross-lagged and autoregressive effects							**C**oncomitant effects						
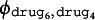	0.467	(***)	0.326	(***)	0.356	(***)	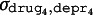	0.111	(***)	0.185	(***)	0.123	(***)
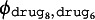	0.444	(***)	0.357	(***)	0.338	(***)	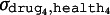	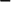 0.198	(***)	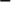 0.411	(***)	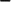 0.268	(***)
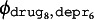	0.103		0.091		0.155	(*)	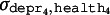	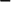 0.209	(***)	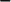 0.369	(***)	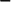 0.186	(***)
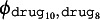	0.382	(***)	0.279	(***)	0.438	(***)	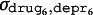	0.238	(**)	0.209	(**)	0.172	(***)
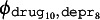	0.106		0.085		0.149	(*)	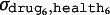	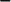 0.360	(***)	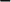 0.325	(***)	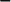 0.234	(***)
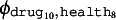	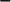 0.192	(***)	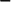 0.135	(***)	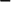 0.241	(***)	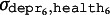	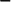 0.323	(***)	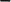 0.309	(***)	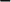 0.165	(***)
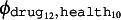	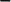 0.094		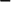 0.072	(*)	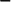 0.095		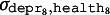	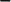 0.225	(***)	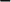 0.206	(***)	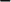 0.099	(***)
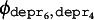	0.048		0.081	(*)	0.077	(*)	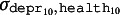	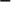 0.153	(***)	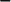 0.135	(***)	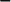 0.065	(***)
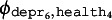	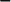 0.213	(***)	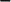 0.133	(***)	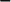 0.123	(***)	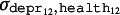	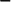 0.336	(***)	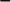 0.288	(***)	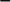 0.134	(***)
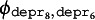	0.115	(**)	0.122	(***)	0.131	(***)	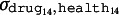	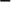 0.181	(*)	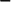 0.096		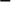 0.068	
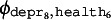	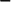 0.170	(***)	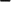 0.153	(***)	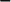 0.174	(***)	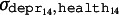	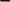 0.515	(***)	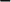 0.484	(***)	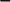 0.246	(***)
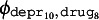	0.087	(*)	0.089	(*)	0.066	(*)							
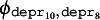	0.163	(***)	0.133	(***)	0.161	(***)							
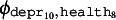	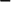 0.238	(***)	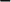 0.215	(***)	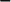 0.220	(***)	**Multivariate linear latent growth component**						
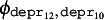	0.166	(***)	0.182	(***)	0.154	(***)							
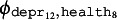	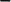 0.216	(***)	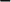 0.210	(***)	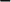 0.225	(***)							
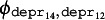	0.132	(**)	0.164	(***)	0.180	(***)	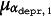	0		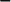 0.089	(***)	0.041	(**)
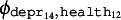	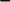 0.334	(**)	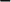 0.314	(***)	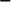 0.292	(***)	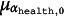	0		0		2.399	(***)
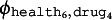	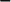 0.132	(**)	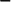 0.093	(**)	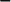 0.067	(**)	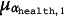	0		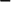 0.091	(***)	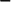 0.183	(***)
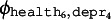	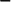 0.261	(**)	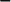 0.218	(**)	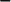 0.205	(**)							
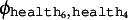	0.090	(**)	0.063	(**)	0.075	(**)							
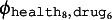	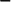 0.160	(***)	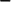 0.145	(***)	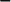 0.102	(***)	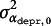	0.571	(***)	0.452	(***)	0.210	(***)
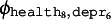	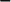 0.243	(***)	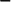 0.240	(***)	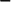 0.249	(***)	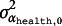	0.939	(***)	0.890	(***)	0.380	(***)
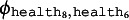	0.099	(***)	0.099	(***)	0.124	(***)							
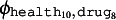	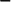 0.111	(***)	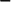 0.125	(***)	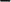 0.098	(***)							
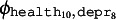	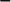 0.241	(***)	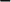 0.262	(***)	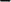 0.246	(***)	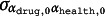	0.323	(**)	0.264	(**)	0.197	(***)
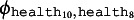	0.177	(***)	0.166	(***)	0.176	(***)	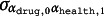	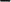 0.060	(*)	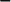 0.038		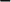 0.027	
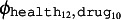	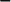 0.069	(*)	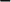 0.100	(*)	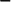 0.059	(*)	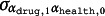	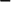 0.062		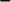 0.053	(*)	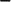 0.042	(**)
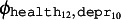	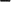 0.266	(***)	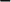 0.297	(***)	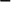 0.288	(***)	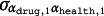	0.026	(*)	0.015	(*)	0.012	(*)
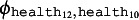	0.177	(***)	0.183	(***)	0.185	(***)	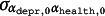	0.388	(***)	0.356	(***)	0.190	(***)
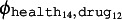	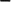 0.105	(**)	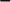 0.169	(**)	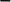 0.112	(**)	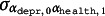	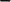 0.105	(***)	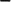 0.096	(***)	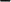 0.050	(***)
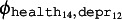	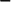 0.334	(***)	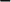 0.357	(***)	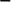 0.362	(***)	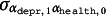	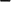 0.102	(***)	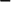 0.092	(***)	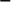 0.049	(***)
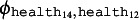	0.211	(***)	0.275	(***)	0.242	(***)	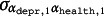	0.049	(***)	0.044	(***)	0.022	(***)
							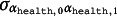	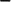 0.067	(*)	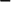 0.059	(*)	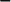 0.019	
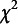	253.187		300.692		266.853								
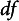	63		84		84								
*p*-value	0.000		0.000		0.000								
CFI (TLI)	0.994	(0.986)	0.994	(0.988)	0.995	(0.990)							
RMSEA	0.024		0.022		0.020								
BIC	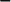 287.174		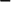 419.789		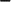 453.629								

Significant estimates are selectively displayed in the table for clarity, and parameters are grouped based on their relevance to either the cross-lagged/autoregressive component of ALT or the multivariate latent growth part. Thresholds and error variances are not reported here due to space constraints but are all found to be significantly different from zero. All models demonstrate a comparable fit to the data, with a superior performance observed for the ALT based on the alternative 2 parameterization.

Examining the cross-lagged relationships among the underlying variables, we consistently find that the perception of health status at each wave is influenced by both the prior perception and the propensities of using illegal drugs and being depressed in the preceding period. The propensity for being depressed on a given occasion is influenced by the propensity at the previous occasion and the prior perception of health status. Notably, the standard parameterization indicates a non-significant autoregressive effect in wave 6.

Divergent conclusions emerge regarding the cross-lagged and autoregressive relationship for the drug variable in different parameterizations. While all models detect a significant influence of prior drug use propensity on the current level during the late adolescence period (waves 4, 6, and 8), additional influences of the prior depressive symptoms propensity are found only in the model based on the alternative 2 parameterization proposed by Mehta et al. (2004).

In the adulthood period (waves 10-12-14), the two alternative parameterizations estimate a significant impact of the previous general health status on the propensity of using drugs, aligning with the findings by Silver et al. (2023).

Consistent conclusions are drawn across all parameterizations in terms of concomitant effects. Regarding the associative multivariate growth component of ALT models, both the growth components (intercept and slope) associated with the underlying variables of depressive symptoms and general health status are correlated.

On the other hand, the intercept and slope associated with the propensity to use drugs correlate with the intercept and slope of the health status variable, respectively. None of the growth components specific to each variable correlate with each other, except for the perception of the health status, but not in the alternative 2 parameterization.

In the standard parameterization, the intercept associated with the drug’s underlying variable correlates with the slope of the general health variable; conversely, in the alternative parameterizations, the slope of the drug variable correlates with the intercept of the health status variable.

Hence, these empirical results underscore the critical impact of alternative parameterizations in autoregressive latent trajectory models on the conclusions that researchers can draw when applying these models to real data.

## 6 Conclusions

In this paper, we have undertaken a thorough examination of how various scaling constraints, implemented to ensure model identification, influence the specification of SEMs used in the analysis of longitudinal categorical data. Our study combines theoretical considerations with empirical validations, providing essential insights into the potential consequences of different parameterization choices.

We focused our attention on ALT models and their special cases: the linear latent growth and first-order autoregressive model. Theoretical investigations have revealed that different parameterizations of the auxiliary model can yield different specifications of the linear latent growth model and of the autoregressive model that, in some cases, are equivalent. Equivalence between alternative specifications has been proven following the approach proposed by Raykov and Penev (1999), such that one-to-one relationships between the parameters of these equivalent specifications have been derived. However, we have shown that when the latent growth model and first-order autoregressive component are jointly considered in the ALT model, different specifications of the auxiliary models imply nonequivalent ALT specifications that are characterized by different constraints on 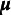 and 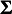 .

The implications of the different nonequivalent parameterizations of the ALT model have been evaluated empirically using data from the NLSY97 cohort in examining the relationship between illegal drug use, depressive symptoms, and general health status. By fitting different specifications of the autoregressive latent trajectory model to these data, temporal influences on health perception emerge as a consistent pattern, revealing significant associations between prior perceptions, propensities for drug use, depressive symptoms, and perceived health status.

The examination of the causes affecting the propensity to use illegal drugs reveals sensitivity to parameterization choices since different specifications can provide us with different answers. Using alternative parametrizations showed us that there are extra factors influencing the propensity of using drugs beyond what the standard model suggests. This emphasizes how researchers need to be careful when picking and imposing different identification constraints. We suggest that the alternative parameterization of the auxiliary model proposed by Jöreskog (2001) be more widely adopted, as it closely aligns with the case of observed continuous variables and provides a more coherent approach than what is often used in practice.

Drawing strength from the comprehensive exploration of both theoretical considerations and empirical validations, our study leverages data from the NLSY97 cohort. This dataset provides a robust empirical foundation, aligning seamlessly with the theoretical framework and enhancing the credibility of our findings. Future research could extend these insights by exploring diverse dataset to validate observed patterns across different populations. Additionally, while our focus on specific models provides depth in understanding these structures, further research is needed to explore the applicability of our findings to other SEM configurations.

In conclusion, this paper contributes valuable insights into the complexities of parameterization choices in SEMs for longitudinal categorical data.

## Supporting information

Bianconcini and Bollen supplementary material Bianconcini and Bollen supplementary material

## Footnotes

1 The WLSMV” estimator is the default estimation option in Mplus 8.6 and lavaan 0.6-16 when some observed (endogenous) variables are categorical. It uses the diagonal of the asymptotic covariance matrix of the sample statistics and the full matrix for inference (standard errors and test statistics), while “MV” refers to Satterthwaite’s mean- and variance-adjusted test statistics (Satterthwaite, 1941).

2 In handling missing data for categorical endogenous variables, lavaan 0.6-16 and Mplus 8.6 use different default strategies. lavaan 0.6-16 uses listwise deletion with the WLSMV estimator. That is, only observations with complete data on all the variables included in the model are used in the analysis. Mplus 8.6 uses, by default, pairwise deletion, meaning that for each pair of variables, only cases with complete data on both variables are used in the estimation. In lavaan 0.6-16, the pairwise deletion can be applied by selecting the option missing=“pairwise” in conjunction with estimator = “WLSMV.” Alternatively, Mplus supports multiple imputation to handle missing data. Based on Bayesian estimation, it creates several imputed data sets using the posterior distribution of the missing data and then pools the results. Lavaan 0.6-16 does not directly perform multiple imputation, but external packages like mice or Amelia should be used, and lavaan 0.6-16 applies to the imputed datasets.

3 When specifying models in Mplus 8.6, it is important to recognize that intercepts and/or underlying variable means need to be specified differently compared to lavaan 0.6-16. In Mplus 8.6, a perfectly measured factor may be introduced behind each  variable to estimate intercepts or underlying variable means. This factor represents the intercept parameters as structural intercepts. In contrast, lavaan 0.6-16 allows for a more straightforward specification of intercepts or means, simplifying the model specification process.

4 An argument could be made that some of the thresholds of an ordinal repeated measure need not be equal over time due to a shift in the meaning of one or more thresholds. However, for our purposes, we maintain the assumption of equal threshold invariance to simplify the discussion.

## Supplementary material

The supplementary material for this article can be found at https://doi.org/10.1017/psy.2024.23.

## Appendix A. One-to-one relationship between alternative parameterizations of the auxiliary model

A one-to-one correspondence between the parameters of the two alternative parameterizations of the auxiliary model can be derived. On the first occasion,

On subsequent occasions,

Conversely, the parameters of the alternative 2 parameterization are functions of those under the alternative 1 specification as follows

## Appendix B. Model identification

### B1. *Linear latent growth models for categorical repeated measures*

Sufficient conditions for the identification of the linear latent growth model for categorical data are discussed here. We consider the three different parameterizations proposed in Section 4.1.1, based on the standard and alternative specifications of the auxiliary model. We also disentangle between the cases in which variance constraints are placed on the underlying variable or on error variances.

***Standard linear latent growth model parameterizations***

Sufficient conditions for the identification of the linear latent growth model based on the standard parameterization of the auxiliary model are

- **Stage 1.**  and diag(  .
- **Stage 2.**  .

In this parameterization, the  parameters to be identified are the time-dependent thresholds,  , and the variance-covariance matrix of the growth components  .

Stage 1 conditions imply the identification of the thresholds on all occasions. The underlying variables are standard normal on each occasion, and estimation for all thresholds are formed as percentiles from the standard normal distribution based on observed frequencies for  , the measured variables. Stage 2 constraints are sufficient for the identification of all the parameters of the growth model (  ). Based on the Stage 1 constraints, the implied mean condition in eq. (13) is satisfied if  , being  a matrix of constant quantities.

The implied polychoric correlations  depend on the growth parameters as follows

The  polychoric correlations are directly estimable, and these known quantities can be used to identify  . As in the presence of continuous data, at least three waves of data (  ) are needed for  to be identified without further restrictions. Indeed, the first three correlations are given by

These equations lead to an expression of the growth parameters,  and  , in terms of identified quantities. That is,  ,  and  . This ensures the identification of  . The error variances  are not parameters but are determined as remainder in eq. (14). That is,  .

When the theta parameterization is adopted, in the Stage 1 conditions diag(  is replaced by  whereas all other constraints remain the same. The proof of identification of this alternative specification begins by considering the expression for the *c*th standardized threshold of  at the first occasion, that is,

where  is the variance of the random intercept. On subsequent occasions,

For the thresholds to be identified, we need to prove the identification of the covariance matrix of the growth components  . We recall that the correlation matrix of the latent response variates can be written as

with  being the diagonal “scaling” matrix in Mplus 8.6, with diagonal elements equal to  .  for the two growth factors can be factorized as

with  a  diagonal matrix,  the  factor covariance matrix, and  a  loading matrix. This factoring is unique given that (  ) ensure a rotational uniqueness. It follows that  , and consequently  , is identified. This identifies diag(  ), and we can identify the full matrix  through rescaling of  . This ensures the identification of  as detailed before. As a consequence, all the thresholds  , are identified based on the equations given above.

***Alternative 1 parameterization of the linear growth model***

Under the alternative parameterization proposed by Muthén and Asparouhov (2002), sufficient conditions for the identification of the linear growth model are:

**Stage 1.** 1.  and  .

2. At all occasions,  .

**Stage 2.** 3.  .

We have  parameters to identify, that is (  ) time-invariant thresholds, the mean of the slope factor, and the three variances and covariance of the growth factors. First, we note that condition 1 ensures the identification of all the thresholds on the first occasion, and their estimates are formed as standard normal percentiles based on the observed frequencies of  . A second consequence of condition 1 is that all the thresholds that are constrained to invariance based on condition 2 are identified by the values on the first occasion. Based on conditions 1 and 2, the means and variances of the underlying variables on subsequent occasions are also identified. To see this, consider the two standardized thresholds that are directly estimable using the observed frequencies of  . They are given by

These are two equations in two unknowns:  and  . We can solve for them, proving their identification. The solutions for  on each occasion immediately lead to a solution for  based on constraint 3 and on the implied mean condition in eq. (13).

Next, the identification of the growth factor covariance matrix  derives directly from the identification of the variances of the underlying variables diag(  ) and their polychoric correlations  . In particular, based on the implied covariance matrix (14) under this parameterization, it can be easily shown that

All the quantities on the left-hand side of each equation are known to be identified. We have three equations in three unknowns,  , and  . We can solve for them, which are then identified. As in the previous case, at least three waves of data (  ) are needed for  to be identified without further restrictions.

An alternative set of constraints is obtained by replacing  with  in condition 1, and allowing the error variances on subsequent occasions to be free parameters. That is,

**Stage 1.** 1.  .

2. At all occasions,  .

**Stage 2.** 3.  .

4.  .

Conditions 1–4 are also sufficient for identification. To understand this part, we recall the Raykov and Penev (1999) relationship that exists between the thresholds and growth parameters under this theta specification and the corresponding parameters under the alternative (delta) parameterization, as detailed in Table 8. Hence, identifying the thresholds and growth parameters based on the theta parameterization follows from identifying the parameters under their delta counterparts.

***Alternative 2 parametrization of the linear growth model***

Under the alternative parameterization proposed by Mehta et al. (2004), the only sufficient condition for the identification of the linear growth model is:

**Stage 1.** 1. At all occasions,  , and  .

This linear growth model specification depends on  thresholds, two means of the growth factors, and their three variances and covariances for a total of  parameters. Condition 1 ensures the identification of the means and variances of  on each occasion. Indeed, the first two standardized thresholds on a given occasion are given by

These are two equations in two unknowns:  and  , that are given by

Similarly, it can be easily shown that also all the thresholds are identified as  The solution for  immediately leads to a solution for  , due to the implied mean condition  , where  is a constant matrix.

Finally, the identification of the growth factor covariance matrix  derives directly from the identification of the underlying variable variances diag(  ) and their polychoric correlations. Based on the implied covariance matrix (14) under this parameterization, it can be easily shown that

All the quantities on the left-hand side of each equation are known to be identified. We have three equations in three unknowns,  and  . We can solve for them, and this ensures their identification. As in the previous case, at least three waves of data (  ) are needed to identify the latter without further restrictions.

When the theta parameterization is adopted, condition 1 on the thresholds is still sufficient for the model identification, which proceeds in a similar manner to that detailed above.

### B2. *Linear latent growth models for binary repeated measures*

The identification of the linear latent growth models for binary data, as reported in Table 10, closely follows the same line outline earlier for categorical data. It is important to take note of the alternative specification suggested by Muthén and Muthén (1998–2017).

***Alternative 1 parametrization by Muthén and Muthén (1998–2017) of the linear growth model***

Under the alternative parameterization proposed by Muthén and Asparouhov (2002), the identification of the linear growth model relies on the following sufficient conditions:

**Stage 1.** 1.  ,  , and  .

2. At all occasions,  .

**Stage 2.** 3.  .

Due to the interdependence of the underlying variable moments  ) on growth parameters  , as detailed in eqs. (13) and (14), we can apply fewer constraints than those specified in Stage 1. This is achieved by simultaneously addressing the identification problem for both the thresholds,  ) and the latent growth parameters  ).

Assuming a standard normal distribution for the underlying variable at the first occasion facilitates the identification of the threshold  . In conjunction with condition 2, all thresholds are identified. For subsequent occasions, considering  , fixing another variance, *e.g.,*  , is sufficient to identify all the means and variances of the underlying variables. Specifically, with the standardized threshold  , the slope mean  is identified. Consequently, based on  , all the variances of the underlying variables are identified.

The proof of identification for  follows a similar approach to the categorical case.

### B3. *Autoregressive of order one models for categorical repeated measures*

Sufficient conditions for the identification of the autoregressive model for categorical data are discussed here. We consider the three different parameterizations proposed in Section 4.1.2 based on the standard and alternative specifications of the auxiliary model. The latter are based on the assumption of threshold invariance. However, if this assumption is relaxed, proofs follow in the same fashion as detailed here.

***Standard (theta) autoregressive model parameterization***

Sufficient conditions for the identification of the autoregressive of order one model based on the standard parameterization of the auxiliary model are

- **Stage 1.** 1.
- **Stage 2.** 2.
  3.  .

In this parameterization, the parameters to be identified are the  time-dependent thresholds,  , and the (  ) autoregressive coefficients  .

On the first occasion, conditions 1 and 2 ensure the identification of all the thresholds  , determined as percentiles of the standard normal based on the observed frequencies of  . On the subsequent occasions, we consider the standardized thresholds, known to be directly estimable, that can be expressed as

where  is the autoregressive coefficient of the relationship of  on  .

The  polychoric correlations are directly estimable, and these known quantities can be used to identify  . Based on this model specification,

such that  results identified. Iterating the procedure, it can be proved that also  is identified. In general, all the autoregressive coefficients can be expressed as

From the identification of the autoregressive coefficients, the identification of the thresholds follows. Hence, the model is identified.

***Alternative 1 parametrization of the autoregressive model***

Under the alternative parameterization proposed by Muthén and Asparouhov (2002), sufficient conditions for the identification of the autoregressive model are:

**Stage 1.** 1.  .

2. At all occasions,  .

**Stage 2.** 3.  .

4.  .

We have  parameters to identify, that is,  ) time-invariant thresholds,  ) intercepts,  ) autoregressive coefficients, and  ) error variances. First, we note that condition 1 and 3 ensures the identification of all the thresholds on the first occasion, and their estimates are formed as standard normal percentiles based on the observed frequencies of  . A second consequence of condition 1 is that all the thresholds, constrained to invariance based on condition 2, are identified by the values on the first occasion. Based on conditions 1–3, the means and variances of the underlying variables on subsequent occasions are also identified. To see this, consider the two standardized thresholds that are directly estimable using the observed frequencies of  . They are given by

These are two equations in two unknowns:  and  . We can solve for them, proving their identification. These known identified variances can be used for identification of the autoregressive coefficients  . Indeed, the polychoric correlations implied by this model specification are such that

That is,  is identified. Furthermore, being  , also  is identified. In general, it follows that

Hence, all autoregressive coefficients are identified. The solution for  and  immediately lead to a solution for  based on the implied mean condition given in eq. (15). Furthermore, based on the implied covariance matrix (16), the identification of  and diag(  ) lead to the identification of  .

***Alternative 2 parametrization of the autoregressive of order one model***

Under the alternative parameterization proposed by Mehta et al. (2004), the only sufficient condition for the identification of the autoregressive model is

**Stage 1.** 1. At all occasions,  , and  .

The parameters to be estimated in this autoregressive model specification are  thresholds, *T* intercepts,  autoregressive coefficients, and *T* error variances. Condition 1 ensures the identification of the means and variances of  on each occasion. Indeed, the first two standardized thresholds on a given occasion are

These are two equations in two unknowns:  and  , that are given by

Similarly, it can be easily shown that also all the thresholds are identified as  Hence, the identification of  , and  proceeds in the same way as detailed for the alternative 1 parameterization.
