# Supplementary material for: Implications of Alternative Parameterizations in Structural Equation Models for Longitudinal Categorical Variables
Source: Psychometrika. 2025 Jan 3;90(2):554–87. doi: 10.1017/psy.2024.23 (PMC12483723; doi:10.1017/psy.2024.23)
Supplement: Bianconcini and Bollen supplementary material [file S0033312324000231sup001.pdf]

Supplementary Material to "Implications of alternative parameterizations in structural equation models for longitudinal categorical variables"

1 Auxiliary models for the NLSY97 data

As discussed in Section 3.1, various parameterizations can be considered for the auxiliary model that links the observed variables to the underlying continuous ones. Both the standard auxiliary model and alternative parameterizations, where no threshold invariance constraints are imposed, result in just identified models (with zero degrees of freedom) that perfectly fit the data. These models can be fitted to the data to obtain estimates of unknown thresholds, means of underlying variables, their variances, and polychoric correlations/covariances among them.

For illustrative purposes, Tables 1 and 2 report the estimated means and polychoric correlations/covariances for the auxiliary model that jointly considers all three observed variables based on the standard and alternative 1 parameterizations with and without threshold invariance. The latter provide similar estimates of the auxiliary model based on the alternative 2 parameterization with and without threshold invariant over time, so they are not reported in this material.

Standard parameterization

Variable	$\mu_{Y_t^*}$	$drug_4$	$drug_6$	$drug_8$	$drug_{10}$	$drug_{12}$	$drug_{14}$	$depr_4$	$depr_6$	$depr_8$	$depr_{10}$	$depr_{12}$	$depr_{14}$	$health_4$	$health_6$	$health_8$	$health_{10}$	$health_{12}$	$health_{14}$
		$\Sigma_{\mathbf{Y}^*\mathbf{Y}^*}$																	
$drug_4$	0	1																	
$drug_6$	0	0.552	1																
$drug_8$	0	0.485	0.659	1															
$drug_{10}$	0	0.433	0.574	0.731	1														
$drug_{12}$	0	0.421	0.513	0.643	0.730	1													
$drug_{14}$	0	0.466	0.437	0.597	0.662	0.738	1												
$depr_4$	0	0.113	0.044	0.044	0.053	0.033	-0.001	1											
$depr_6$	0	0.087	0.154	0.090	0.151	0.104	0.068	0.458	1										
$depr_8$	0	0.029	0.055	0.144	0.145	0.100	0.086	0.417	0.469	1									
$depr_{10}$	0	0.069	0.089	0.130	0.242	0.122	0.163	0.387	0.442	0.521	1								
$depr_{12}$	0	0.026	-0.005	0.067	0.120	0.169	0.119	0.375	0.407	0.468	0.516	1							
$depr_{14}$	0	0.057	0.021	0.093	0.121	0.143	0.200	0.313	0.383	0.432	0.501	0.558	1						
$health_4$	0	-0.200	-0.110	-0.043	-0.095	-0.033	-0.000	-0.210	-0.174	-0.149	-0.172	-0.129	-0.163	1					
$health_6$	0	-0.123	-0.150	-0.038	-0.070	-0.055	-0.050	-0.197	-0.229	-0.182	-0.195	-0.163	-0.196	0.548	1				
$health_8$	0	-0.144	-0.099	-0.091	-0.170	-0.090	-0.029	-0.167	-0.204	-0.245	-0.220	-0.183	-0.179	0.503	0.564	1			
$health_{10}$	0	-0.077	-0.063	-0.039	-0.130	-0.083	-0.048	-0.160	-0.193	-0.188	-0.242	-0.180	-0.185	0.444	0.515	0.580	1		
$health_{12}$	0	-0.090	-0.047	-0.025	-0.088	-0.062	-0.063	-0.179	-0.194	-0.192	-0.208	-0.251	-0.218	0.441	0.503	0.525	0.565	1	
$health_{14}$	0	-0.063	-0.049	-0.027	-0.116	-0.112	-0.097	-0.171	-0.190	-0.183	-0.206	-0.210	-0.290	0.389	0.449	0.507	0.535	0.602	1

Alternative 1 parameterization

Variable	$\mu_{Y_t^*}$	$drug_4$	$drug_6$	$drug_8$	$drug_{10}$	$drug_{12}$	$drug_{14}$	$depr_4$	$depr_6$	$depr_8$	$depr_{10}$	$depr_{12}$	$depr_{14}$	$health_4$	$health_6$	$health_8$	$health_{10}$	$health_{12}$	$health_{14}$
		$\Sigma_{\mathbf{Y}^*\mathbf{Y}^*}$																	
$drug_4$	0.000	1																	
$drug_6$	-0.051	0.552	1																
$drug_8$	-0.098	0.485	0.659	1															
$drug_{10}$	-0.169	0.433	0.574	0.731	1														
$drug_{12}$	-0.269	0.421	0.513	0.643	0.730	1													
$drug_{14}$	-0.379	0.466	0.437	0.597	0.662	0.738	1												
$depr_4$	0.000	0.113	0.044	0.044	0.053	0.033	-0.001	1											
$depr_6$	-0.003	0.087	0.154	0.090	0.150	0.104	0.068	0.457	1.002										
$depr_8$	-0.141	0.029	0.056	0.145	0.146	0.101	0.087	0.420	0.473	0.991									
$depr_{10}$	-0.238	0.070	0.090	0.131	0.244	0.123	0.165	0.390	0.445	0.530	0.992								
$depr_{12}$	-0.217	0.025	-0.005	0.066	0.118	0.166	0.118	0.369	0.400	0.465	0.513	1.015							
$depr_{14}$	-0.266	0.057	0.021	0.093	0.121	0.143	0.199	0.312	0.381	0.434	0.503	0.548	1.004						
$health_4$	0.000	-0.200	-0.110	-0.043	-0.095	-0.033	-0.000	-0.210	-0.174	-0.150	-0.173	-0.127	-0.162	1					
$health_6$	-0.206	-0.113	-0.137	-0.035	-0.064	-0.051	-0.045	-0.180	-0.209	-0.168	-0.179	-0.147	-0.178	0.501	1.094				
$health_8$	-0.351	-0.119	-0.082	-0.075	-0.141	-0.074	-0.024	-0.138	-0.169	-0.204	-0.183	-0.149	-0.148	0.416	0.426	1.209			
$health_{10}$	-0.291	-0.069	-0.057	-0.035	-0.117	-0.075	-0.043	-0.144	-0.174	-0.171	-0.219	-0.160	-0.166	0.399	0.424	0.432	1.112		
$health_{12}$	0.338	-0.080	-0.042	-0.022	-0.079	-0.055	-0.056	-0.161	-0.174	-0.173	-0.188	-0.222	-0.195	0.396	0.413	0.390	0.456	1.115	
$health_{14}$	-0.393	-0.059	-0.046	-0.026	-0.111	-0.107	-0.092	-0.163	-0.180	-0.175	-0.197	-0.197	-0.275	0.370	0.390	0.399	0.457	0.514	1.051

Table 1: Estimated mean vector and correlation/covariance matrix for the underlying variables $\mathbf{Y}_i^*$ based on the standard and alternative 1 (no threshold invariance) parameterizations of the auxiliary model.

______: not significant at 5% level.

Alternative 1 parameterization - threshold invariance

Variable	$\mu_{Y_t^*}$	$\mathbf{\Sigma}_{\mathbf{Y}^*\mathbf{Y}^*}$																	
		$drug_4$	$drug_6$	$drug_8$	$drug_{10}$	$drug_{12}$	$drug_{14}$	$depr_4$	$depr_6$	$depr_8$	$depr_{10}$	$depr_{12}$	$depr_{14}$	$health_4$	$health_6$	$health_8$	$health_{10}$	$health_{12}$	$health_{14}$
$drug_4$	0.000	1																	
$drug_6$	-0.051	0.552	1																
$drug_8$	-0.098	0.485	0.659	1															
$drug_{10}$	-0.169	0.433	0.574	0.731	1														
$drug_{12}$	-0.269	0.421	0.513	0.643	0.730	1													
$drug_{14}$	-0.379	0.466	0.437	0.597	0.662	0.738	1												
$depr_4$	0.000	0.113	0.044	0.044	0.053	0.033	-0.001	1											
$depr_6$	-0.012	0.088	0.156	0.091	0.153	0.105	0.068	0.464	0.986										
$depr_8$	-0.138	0.029	0.056	0.144	0.145	0.100	0.087	0.419	0.478	0.995									
$depr_{10}$	-0.241	0.070	0.090	0.132	0.244	0.123	0.165	0.391	0.453	0.529	0.990								
$depr_{12}$	-0.238	0.026	-0.005	0.068	0.122	0.171	0.121	0.380	0.419	0.477	0.530	0,985							
$depr_{14}$	-0.291	0.059	0.021	0.096	0.125	0.148	0.205	0.322	0.400	0.447	0.521	0.583	0.972						
$health_4$	0.000	-0.200	-0.110	-0.043	-0.095	-0.033	-0.000	-0.210	-0.174	-0.149	-0.174	-0.131	-0.167	1					
$health_6$	-0.120	-0.116	-0.142	-0.036	-0.067	-0.052	-0.047	-0.187	-0.229	-0.173	-0.186	-0.157	-0.191	0.518	1.057				
$health_8$	-0.158	-0.134	-0.092	-0.085	-0.158	-0.083	-0.027	-0.155	-0.192	-0.228	-0.206	-0.172	-0.171	0.466	0.494	1.079			
$health_{10}$	-0.198	-0.072	-0.059	-0.037	-0.121	-0.078	-0.045	-0.150	-0.184	-0.188	-0.229	-0.171	-0.178	0.415	0.456	0.503	1.068		
$health_{12}$	-0.286	-0.082	-0.043	-0.023	-0.080	-0.056	-0.057	-0.164	-0.179	-0.175	-0.191	-0.232	-0.204	0.402	0.434	0.444	0.482	1.097	
$health_{14}$	-0.339	-0.060	-0.047	-0.026	-0.111	-0.108	-0.093	-0.164	-0.185	-0.176	-0.200	-0.204	-0.286	0.373	0.407	0.451	0.480	0.527	1.042

Table 2: Estimated mean vector and correlation/covariance matrix for the underlying variables $\mathbf{Y}_i^*$ based on the alternative 1 (with threshold invariance) parameterization of the auxiliary model.

______: not significant at 5% level.

2 Linear latent growth models for categorical variables - implied moments

The corresponding implied moments has to be derived to prove the equivalence of alternative specifications of the linear latent growth models.

When the standard parameterization is adopted for the auxiliary model, $(\boldsymbol{\mu}_{\mathbf{Y}^*}, \texttt{diag}(\boldsymbol{\Sigma}_{\mathbf{Y}^*\mathbf{Y}^*}))$ is set to $(\mathbf{0}, \mathbf{I})$ and thresholds are assumed to be time-varying $\tau_{ct}^{std}, c = 1, \ldots, C-1$, $t = 1, \ldots, T$. As described in the paper, an alternative set of identification conditions is derived by replacing $\texttt{diag}(\boldsymbol{\Sigma}_{\mathbf{Y}^*\mathbf{Y}^*}) = \mathbf{I}$ with $\boldsymbol{\Theta}_{\boldsymbol{\varepsilon}} = \mathbf{I}$. The mean vector of the underlying variables implied by the linear growth model is always a zero vector $\boldsymbol{\mu}'_{\mathbf{Y}^*} = [0 \quad 0 \quad 0 \quad 0]$. On the other hand, the covariance matrix implied by fixing $\texttt{diag}(\boldsymbol{\Sigma}_{\mathbf{Y}^*\mathbf{Y}^*}) = \mathbf{I}$ is

$$\boldsymbol{\Sigma}_{\mathbf{Y}^*\mathbf{Y}^*} = \begin{bmatrix} 1 & & & \\ \sigma^2_{\alpha_0} + \sigma_{\alpha_0,\alpha_1} & 1 & & \\ \sigma^2_{\alpha_0} + 2\sigma_{\alpha_0,\alpha_1} & \sigma^2_{\alpha_0} + 3\sigma_{\alpha_0,\alpha_1} + 2\sigma^2_{\alpha_1} & 1 & \\ \sigma^2_{\alpha_0} + 3\sigma_{\alpha_0,\alpha_1} & \sigma^2_{\alpha_0} + 4\sigma_{\alpha_0,\alpha_1} + 3\sigma^2_{\alpha_1} & \sigma^2_{\alpha_0} + 5\sigma_{\alpha_0,\alpha_1} + 6\sigma^2_{\alpha_1} & 1 \end{bmatrix}$$

whereas, when $\boldsymbol{\Theta}_{\boldsymbol{\varepsilon}} = \mathbf{I}$,

$$\boldsymbol{\Sigma}_{\mathbf{Y}^*\mathbf{Y}^*} = \begin{bmatrix} \sigma^2_{\alpha_0} + 1 & & & \\ \sigma^2_{\alpha_0} + \sigma_{\alpha_0,\alpha_1} & \sigma^2_{\alpha_0} + 2\sigma_{\alpha_0,\alpha_1} + \sigma^2_{\alpha_1} + 1 & & \\ \sigma^2_{\alpha_0} + 2\sigma_{\alpha_0,\alpha_1} & \sigma^2_{\alpha_0} + 3\sigma_{\alpha_0,\alpha_1} + 2\sigma^2_{\alpha_1} & \sigma^2_{\alpha_0} + 4\sigma_{\alpha_0,\alpha_1} + 4\sigma^2_{\alpha_1} + 1 & \\ \sigma^2_{\alpha_0} + 3\sigma_{\alpha_0,\alpha_1} & \sigma^2_{\alpha_0} + 4\sigma_{\alpha_0,\alpha_1} + 3\sigma^2_{\alpha_1} & \sigma^2_{\alpha_0} + 5\sigma_{\alpha_0,\alpha_1} + 6\sigma^2_{\alpha_1} & \sigma^2_{\alpha_0} + 6\sigma_{\alpha_0,\alpha_1} + 9\sigma^2_{\alpha_1} + 1 \end{bmatrix}$$

When all variances are freely estimated except on the first occasion, the auxiliary model has at least two time-invariant threshold parameters $\tau_{ct}^{alt1} = \tau_c^{alt1}, c = 1, 2$. This requires the underlying variable at the first occasion to follow a standard normal density, but at the subsequent occasion, Y^*_{it} follows a more general distribution with a mean equal to $\mu^{alt1}_{Y^*_t}$ and variance $\sigma^{2alt1}_{Y^*_t}$. Hence, the implied mean vector of the underlying variables is given by $\boldsymbol{\mu}'_{\mathbf{Y}^*} = [0 \quad \mu_{\alpha_1} \quad 2\mu_{\alpha_1} \quad 3\mu_{\alpha_1}]$, whereas the covariance matrix implied by focusing on the

underlying variable variances results

$$\boldsymbol{\Sigma}_{\mathbf{Y}^*\mathbf{Y}^*} = \begin{bmatrix} 1 & & & \\ \sigma^2_{\alpha_0} + \sigma_{\alpha_0,\alpha_1} & \sigma^{2alt1}_{Y_2^*} & & \\ \sigma^2_{\alpha_0} + 2\sigma_{\alpha_0,\alpha_1} & \sigma^2_{\alpha_0} + 3\sigma_{\alpha_0,\alpha_1} + 2\sigma^2_{\alpha_1} & \sigma^{2alt1}_{Y_3^*} & \\ \sigma^2_{\alpha_0} + 3\sigma_{\alpha_0,\alpha_1} & \sigma^2_{\alpha_0} + 4\sigma_{\alpha_0,\alpha_1} + 3\sigma^2_{\alpha_1} & \sigma^2_{\alpha_0} + 5\sigma_{\alpha_0,\alpha_1} + 6\sigma^2_{\alpha_1} & \sigma^{2alt1}_{Y_4^*} \end{bmatrix}.$$

When the error variances are all free except the one at the first occasion,

$$\boldsymbol{\Sigma}_{\mathbf{Y}^*\mathbf{Y}^*} = \begin{bmatrix} \sigma^2_{\alpha_0} + 1 & & & \\ \sigma^2_{\alpha_0} + \sigma_{\alpha_0,\alpha_1} & \sigma^2_{\alpha_0} + 2\sigma_{\alpha_0,\alpha_1} + \sigma^2_{\alpha_1} + \sigma^2_{\varepsilon_2} & & \\ \sigma^2_{\alpha_0} + 2\sigma_{\alpha_0,\alpha_1} & \sigma^2_{\alpha_0} + 3\sigma_{\alpha_0,\alpha_1} + 2\sigma^2_{\alpha_1} & \sigma^2_{\alpha_0} + 4\sigma_{\alpha_0,\alpha_1} + 4\sigma^2_{\alpha_1} + +\sigma^2_{\varepsilon_3} & \\ \sigma^2_{\alpha_0} + 3\sigma_{\alpha_0,\alpha_1} & \sigma^2_{\alpha_0} + 4\sigma_{\alpha_0,\alpha_1} + 3\sigma^2_{\alpha_1} & \sigma^2_{\alpha_0} + 5\sigma_{\alpha_0,\alpha_1} + 6\sigma^2_{\alpha_1} & \sigma^2_{\alpha_0} + 6\sigma_{\alpha_0,\alpha_1} + 9\sigma^2_{\alpha_1} + +\sigma^2_{\varepsilon_4} \end{bmatrix}$$

Finally, when the Joreskog (2001) parameterization is considered, the first and second thresholds at each occasion are always fixed to zero and one, respectively, whereas the other thresholds $\tau^{alt2}_{ct}, c \geq 3, t = 1, \ldots, T$, are freely estimated. This threshold specification allows the mean and variance of the underlying variable to be freely estimated on each occasion. The implied mean vector of the underlying variables results $\boldsymbol{\mu}'_{\mathbf{Y}^*} = [\mu_{\alpha_0} \quad \mu_{\alpha_0} + \mu_{\alpha_1} \quad \mu_{\alpha_0} + 2\mu_{\alpha_1} \quad \mu_{\alpha_0} + 3\mu_{\alpha_1}]$, whereas the covariance matrix implied by focusing on the underlying variable variances is given by

$$\boldsymbol{\Sigma}_{\mathbf{Y}^*\mathbf{Y}^*} = \begin{bmatrix} \sigma^{2alt2}_{Y_1^*} & & & \\ \sigma^2_{\alpha_0} + \sigma_{\alpha_0,\alpha_1} & \sigma^{2alt2}_{Y_2^*} & & \\ \sigma^2_{\alpha_0} + 2\sigma_{\alpha_0,\alpha_1} & \sigma^2_{\alpha_0} + 3\sigma_{\alpha_0,\alpha_1} + 2\sigma^2_{\alpha_1} & \sigma^{2alt2}_{Y_3^*} & \\ \sigma^2_{\alpha_0} + 3\sigma_{\alpha_0,\alpha_1} & \sigma^2_{\alpha_0} + 4\sigma_{\alpha_0,\alpha_1} + 3\sigma^2_{\alpha_1} & \sigma^2_{\alpha_0} + 5\sigma_{\alpha_0,\alpha_1} + 6\sigma^2_{\alpha_1} & \sigma^{2alt2}_{Y_4^*} \end{bmatrix}.$$

On the other hand, when the error variances are all freely estimated,

$$\boldsymbol{\Sigma}_{\mathbf{Y}^*\mathbf{Y}^*} = \begin{bmatrix} \sigma^2_{\alpha_0} + \sigma^2_{\varepsilon_1} & & & \\ \sigma^2_{\alpha_0} + \sigma_{\alpha_0,\alpha_1} & \sigma^2_{\alpha_0} + 2\sigma_{\alpha_0,\alpha_1} + \sigma^2_{\alpha_1} + \sigma^2_{\varepsilon_2} & & \\ \sigma^2_{\alpha_0} + 2\sigma_{\alpha_0,\alpha_1} & \sigma^2_{\alpha_0} + 3\sigma_{\alpha_0,\alpha_1} + 2\sigma^2_{\alpha_1} & \sigma^2_{\alpha_0} + 4\sigma_{\alpha_0,\alpha_1} + 4\sigma^2_{\alpha_1} + +\sigma^2_{\varepsilon_3} & \\ \sigma^2_{\alpha_0} + 3\sigma_{\alpha_0,\alpha_1} & \sigma^2_{\alpha_0} + 4\sigma_{\alpha_0,\alpha_1} + 3\sigma^2_{\alpha_1} & \sigma^2_{\alpha_0} + 5\sigma_{\alpha_0,\alpha_1} + 6\sigma^2_{\alpha_1} & \sigma^2_{\alpha_0} + 6\sigma_{\alpha_0,\alpha_1} + 9\sigma^2_{\alpha_1} + +\sigma^2_{\varepsilon_4} \end{bmatrix}$$

3 Autoregressive model of order one for categorical variables - implied moments

When standard (theta) parameterization is adopted, $\boldsymbol{\mu}_{Y^*} = \mathbf{0}$, $\boldsymbol{\Theta}_{\boldsymbol{\varepsilon}} = \mathbf{I}$, whereas the thresholds parameters, $\tau_{ct}^{std}, c = 1, \ldots, C-1, t = 1, \ldots, T$, are time-varying. The implied mean vector of the underlying variables is a zero vector $\boldsymbol{\mu}'_{\mathbf{Y}^*} = [0 \quad 0 \quad 0 \quad 0]$. The implied covariance matrix is given by

$$\boldsymbol{\Sigma}_{\mathbf{Y}^*\mathbf{Y}^*} = \begin{bmatrix} 1 & & & \\ \phi_{21} & 1+\phi_{21}^2 & & \\ \phi_{32}\phi_{21} & \phi_{32}(1+\phi_{21}^2) & 1+\phi_{32}^2(1+\phi_{21}^2) & \\ \phi_{43}\phi_{32}\phi_{21} & \phi_{43}\phi_{32}(1+\phi_{21}^2) & \phi_{43}(1+\phi_{32}^2(1+\phi_{21}^2)) & 1+\phi_{43}^2(1+\phi_{32}^2(1+\phi_{21}^2) \end{bmatrix}.$$

When all variances are freely estimated, but on the first occasion, the auxiliary model has at least two time-invariant threshold parameters $\tau_{ct}^{alt1} = \tau_c^{alt1}, c = 1, 2$. This requires the underlying variable at the first occasion to follow a standard normal density, but on the subsequent occasion, it follows a more general distribution with a mean equal to $\mu_{Y_t^*}^{alt1}$ and variance $\sigma_{Y_t^*}^{2alt1}$. Hence, the implied mean vector of the underlying variables is given by $\boldsymbol{\mu}'_{\mathbf{Y}^*} = \begin{bmatrix} 0 & \nu_{Y_2^*} & \nu_{Y_3^*} + \phi_{32}\nu_{Y_2^*} & \nu_{Y_4^*} + \phi_{43}(\nu_{Y_3^*} + \phi_{32}\nu_{Y_2^*}) \end{bmatrix}$, whereas the covariance matrix results

$$\boldsymbol{\Sigma}_{\mathbf{YY}} = \begin{bmatrix} 1 & & & \\ \phi_{21} & \sigma_{\varepsilon_2}^2+\phi_{21}^2 & & \\ \phi_{32}\phi_{21} & \phi_{32}(\sigma_{\varepsilon_2}^2+\phi_{21}^2) & \sigma_{\varepsilon_3}^2+\phi_{32}^2(\sigma_{\varepsilon_2}^2+\phi_{21}^2) & \\ \phi_{43}\phi_{32}\phi_{21} & \phi_{43}\phi_{32}(\sigma_{\varepsilon_2}^2+\phi_{21}^2) & \phi_{43}(\sigma_{\varepsilon_3}^2+\phi_{32}^2(\sigma_{\varepsilon_2}^2+\phi_{21}^2)) & \sigma_{\varepsilon_4}^2+\phi_{43}^2(\sigma_{\varepsilon_3}^2+\phi_{32}^2(\sigma_{\varepsilon_2}^2+\phi_{21}^2) \end{bmatrix}.$$

Finally, when all error variances are freely estimated, the first and second thresholds at each occasion are always fixed to zero and one, respectively, whereas the other thresholds $\tau_{ct}^{alt2}, c \geq 3, t = 1, \ldots, T$ are freely estimated. This threshold specification allows the mean and variance of the underlying variable to be free on each occasion. The implied mean vec-

tor of the underlying variables results $\boldsymbol{\mu}'_{\mathbf{Y}} = \big[\nu_{Y_1^*} \quad \nu_{Y_2^*} + \phi_{21}\nu_{Y_1^*} \quad \nu_{Y_3^*} + \phi_{32}(\nu_{Y_2^*} + \phi_{21}\nu_{Y_1^*})$ $\nu_{Y_4^*} + \phi_{43}(\nu_{Y_3^*} + \phi_{32}(\nu_{Y_2^*} + \phi_{21}\nu_{Y_1^*}))\big]$, whereas the covariance matrix implied by focusing on the error variances is given by

$$\boldsymbol{\Sigma}_{\mathbf{YY}} = \begin{bmatrix} \sigma^2_{\varepsilon_1} & & & \\ \phi_{21} & \sigma^2_{\varepsilon_2} + \phi^2_{21}\sigma^2_{\varepsilon_1} & & \\ \phi_{32}\phi_{21} & \phi_{32}(\sigma^2_{\varepsilon_2} + \phi^2_{21}\sigma^2_{\varepsilon_1}) & \sigma^2_{\varepsilon_3} + \phi^2_{32}(\sigma^2_{\varepsilon_2} + \phi^2_{21}\sigma^2_{\varepsilon_1}) & \\ \phi_{43}\phi_{32}\phi_{21} & \phi_{43}\phi_{32}(\sigma^2_{\varepsilon_2} + \phi^2_{21}\sigma^2_{\varepsilon_1}) & \phi_{43}(\sigma^2_{\varepsilon_3} + \phi^2_{32}(\sigma^2_{\varepsilon_2} + \phi^2_{21}\sigma^2_{\varepsilon_1})) & \sigma^2_{\varepsilon_4} + \phi^2_{43}(\sigma^2_{\varepsilon_3} + \phi^2_{32}(\sigma^2_{\varepsilon_2} + \phi^2_{21}\sigma^2_{\varepsilon_1}) \end{bmatrix}.$$

4 Autoregressive latent trajectory model for categorical variables - implied moments

To avoid tedious and unnecessary complex computations, the moments implied by the different specifications of the autoregressive latent trajectory model are derived assuming a stationary autoregressive component and considering four observed occasions. The implied moments (mean vector and covariance matrix) are detailed for the parameterizations discussed in Section 4.1.3. We recall that the thresholds are assumed to be time-invariant for the alternative parameterizations.

Standard parameterization

$$\boldsymbol{\mu}_{\mathbf{Y}^*} = \mathbf{0}$$

∞

$$\boldsymbol{\Sigma}_{\mathbf{Y}^*\mathbf{Y}^*} = \begin{bmatrix} 1 & & & \\ \sigma_{Y_1^*\alpha_0} + \phi & \sigma^2_{\alpha_0} + \phi\sigma_{Y_1^*\alpha_0} + \phi\sigma_{Y_1^*Y_2^*} + 1 & & \\ \sigma_{Y_1^*\alpha_0} + \sigma_{Y_1^*\alpha_1} + \phi\sigma_{Y_1^*Y_2^*} & \sigma^2_{\alpha_0} + \sigma_{\alpha_0\alpha_1} + \phi(\sigma_{Y_1^*\alpha_0} + \sigma_{Y_1^*\alpha_1}) + \phi\sigma^2_{Y_2^*} & (1+\phi)\sigma^2_{\alpha_0} + (2+\phi)\sigma_{\alpha_0\alpha_1} + \phi^2(\sigma_{Y_1^*\alpha_0} + \sigma_{Y_1^*\alpha_1}) + \sigma^2_{\alpha_1} + \phi\sigma_{Y_2^*Y_3^*} + 1 & \\ \sigma_{Y_1^*\alpha_0} + 2\sigma_{Y_1^*\alpha_1} + \phi\sigma_{Y_1^*Y_3^*} & \sigma^2_{\alpha_0} + 2\sigma_{\alpha_0\alpha_1} + \phi(\sigma_{Y_1^*\alpha_0} + 2\sigma_{Y_1^*\alpha_1}) + \phi\sigma_{Y_2^*Y_3^*} & (1+\phi)\sigma^2_{\alpha_0} + (3+2\phi)\sigma_{\alpha_0\sigma_1} + \phi^2(\sigma_{Y_1^*\alpha_0} + 2\sigma_{Y_1^*\alpha_1}) + 2\sigma^2_{\alpha_1} + \phi\sigma^2_{Y_3^*} & \begin{array}{c}(1+\phi+\phi^2)\sigma^2_{\alpha_0} + (4+3\phi+2\phi^2)\sigma_{\alpha_0\alpha_1} \\ +\phi^3(\sigma_{Y_1^*\alpha_0} + 2\sigma_{Y_1^*\alpha_1}) + (4+2\phi)\sigma^2_{\alpha_1} + \phi\sigma_{Y_3^*Y_4^*} + 1\end{array} \end{bmatrix}$$

Alternative 1 parameterization

$$\boldsymbol{\mu}'_{\mathbf{Y}^*} = \begin{bmatrix} \mu_{Y_1^*} & 0 & \mu_{\alpha_1} + \phi^2\mu_{Y_1^*} & 2\mu_{\alpha_1} + \phi^2\mu_{Y_1^*} \end{bmatrix}$$

$$\boldsymbol{\Sigma}_{\mathbf{Y}^*\mathbf{Y}^*} = \begin{bmatrix} \sigma^2_{\varepsilon_1} & & & \\ \sigma_{Y_1^*\alpha_0} + \phi & \sigma^2_{\alpha_0} + \phi\sigma_{Y_1^*\alpha_0} + \phi\sigma_{Y_1^*Y_2^*} + 1 & & \\ \sigma_{Y_1^*\alpha_0} + \sigma_{Y_1^*\alpha_1} + \phi\sigma_{Y_1^*Y_2^*} & \sigma^2_{\alpha_0} + \sigma_{\alpha_0\alpha_1} + \phi(\sigma_{Y_1^*\alpha_0} + \sigma_{Y_1^*\alpha_1}) + \phi\sigma^2_{Y_2^*} & (1+\phi)\sigma^2_{\alpha_0} + (2+\phi)\sigma_{\alpha_0\alpha_1} + \phi^2(\sigma_{Y_1^*\alpha_0} + \sigma_{Y_1^*\alpha_1}) + \sigma^2_{\alpha_1} + \phi\sigma_{Y_2^*Y_3^*} + \sigma^2_{\varepsilon_3} & \\ \sigma_{Y_1^*\alpha_0} + 2\sigma_{Y_1^*\alpha_1} + \phi\sigma_{Y_1^*Y_3^*} & \sigma^2_{\alpha_0} + 2\sigma_{\alpha_0\alpha_1} + \phi(\sigma_{Y_1^*\alpha_0} + 2\sigma_{Y_1^*\alpha_1}) + \phi\sigma_{Y_2^*Y_3^*} & (1+\phi)\sigma^2_{\alpha_0} + (3+2\phi)\sigma_{\alpha_0\sigma_1} + \phi^2(\sigma_{Y_1^*\alpha_0} + 2\sigma_{Y_1^*\alpha_1}) + 2\sigma^2_{\alpha_1} + \phi\sigma^2_{Y_3^*} & \begin{array}{c}(1+\phi+\phi^2)\sigma^2_{\alpha_0} + (4+3\phi+2\phi^2)\sigma_{\alpha_0\alpha_1} + \phi^3\Big(\sigma_{Y_1^*\alpha_0} \\ +2\sigma_{Y_1^*\alpha_1}\Big) + (4+2\phi)\sigma^2_{\alpha_1} + \phi\sigma_{Y_3^*Y_4^*} + \sigma^2_{\varepsilon_4}\end{array} \end{bmatrix}$$

Alternative 2 parameterization

$$\boldsymbol{\mu}'_{\mathbf{Y}^*} = \begin{bmatrix} \mu_{Y_1^*} & \mu_{\alpha_0} + \phi\mu_{Y_1^*} & \mu_{\alpha_0} + \mu_{\alpha_1} + \phi^2\mu_{Y_1^*} & \mu_{\alpha_0} + 2\mu_{\alpha_1} + \phi^2\mu_{Y_3^*} \end{bmatrix}$$

$$\boldsymbol{\Sigma}_{\mathbf{Y}^*\mathbf{Y}^*} = \begin{bmatrix} \sigma^2_{\varepsilon_1} & & & \\ \sigma_{Y_1^*\alpha_0} + \phi & \sigma^2_{\alpha_0} + \phi\sigma_{Y_1^*\alpha_0} + \phi\sigma_{Y_1^*Y_2^*} + \sigma^2_{\varepsilon_2} & & \\ \sigma_{Y_1^*\alpha_0} + \sigma_{Y_1^*\alpha_1} + \phi\sigma_{Y_1^*Y_2^*} & \sigma^2_{\alpha_0} + \sigma_{\alpha_0\alpha_1} + \phi(\sigma_{Y_1^*\alpha_0} + \sigma_{Y_1^*\alpha_1}) + \phi\sigma^2_{Y_2^*} & (1+\phi)\sigma^2_{\alpha_0} + (2+\phi)\sigma_{\alpha_0\alpha_1} + \phi^2(\sigma_{Y_1^*\alpha_0} + \sigma_{Y_1^*\alpha_1}) + \sigma^2_{\alpha_1} + \phi\sigma_{Y_2^*Y_3^*} + \sigma^2_{\varepsilon_3} & \\ \sigma_{Y_1^*\alpha_0} + 2\sigma_{Y_1^*\alpha_1} + \phi\sigma_{Y_1^*Y_3^*} & \sigma^2_{\alpha_0} + 2\sigma_{\alpha_0\alpha_1} + \phi(\sigma_{Y_1^*\alpha_0} + 2\sigma_{Y_1^*\alpha_1}) + \phi\sigma_{Y_2^*Y_3^*} & (1+\phi)\sigma^2_{\alpha_0} + (3+2\phi)\sigma_{\alpha_0\sigma_1} + \phi^2(\sigma_{Y_1^*\alpha_0} + 2\sigma_{Y_1^*\alpha_1}) + 2\sigma^2_{\alpha_1} + \phi\sigma^2_{Y_3^*} & (1+\phi+\phi^2)\sigma^2_{\alpha_0} + (4+3\phi+2\phi^2)\sigma_{\alpha_0\alpha_1} + \phi^3\Big(\sigma_{Y_1^*\alpha_0} \\ & & & +2\sigma_{Y_1^*\alpha_1}\Big) + (4+2\phi)\sigma^2_{\alpha_1} + \phi\sigma_{Y_3^*Y_4^*} + \sigma^2_{\varepsilon_4} \end{bmatrix}$$

5 Autoregressive latent trajectory model for categorical variables - Σ-constraints

In this Section, we provide the $\boldsymbol{\Sigma}$ constraints for each of the considered specifications of the autoregressive trajectory model. To avoid unnecessary complexity, we still consider a stationary autoregressive of order one process and five observed occasions.

Standard parameterization

$$\begin{aligned}
\sigma^2_{Y_1^*} &= 1 \\
\sigma^2_{Y_2^*} &= \frac{1 + \sigma^2_{Y_2^*Y_1^*} + \sigma_{Y_3^*Y_1^*} + 2\sigma_{Y_3^*Y_2^*} + \sigma_{Y_3^*Y_2^*}\sigma_{Y_4^*Y_1^*} - \sigma_{Y_4^*Y_2^*} - \sigma_{Y_3^*Y_1^*}\sigma_{Y_4^*Y_2^*} + \sigma_{Y_2^*Y_1^*}(-2 - 2\sigma_{Y_3^*Y_1^*} - 3\sigma_{Y_3^*Y_2^*} + \sigma_{Y_4^*Y_1^*} + 2\sigma_{Y_4^*Y_2^*})}{1 - 3\sigma_{Y_3^*Y_1^*} + 2\sigma_{Y_4^*Y_1^*}} \\
\sigma^2_{Y_3^*} &= -\frac{2\sigma^2_{Y_3^*Y_1^*} + \sigma_{Y_3^*Y_2^*} - 2\sigma_{Y_4^*Y_1^*} + 2\sigma_{Y_3^*Y_2^*}\sigma_{Y_4^*Y_1^*} + \sigma_{Y_4^*Y_3^*} + \sigma_{Y_3^*Y_1^*}(4 - 3\sigma_{Y_3^*Y_2^*} - \sigma_{Y_4^*Y_1^*} + \sigma_{Y_4^*Y_3^*}) - \sigma_{Y_2^*Y_1^*}(2 + \sigma_{Y_3^*Y_1^*} + 2\sigma_{Y_4^*Y_3^*})}{-2 + 3\sigma_{Y_2^*Y_1^*} - \sigma_{Y_4^*Y_1^*}} \\
\sigma^2_{Y_4^*} &= \frac{1}{(1 - 2\sigma_{Y_2^*Y_1^*} + \sigma_{Y_3^*Y_1^*})^2}\Big(-2\sigma_{Y_4^*Y_1^*} + 2\sigma^2_{Y_4^*Y_1^*} - \sigma_{Y_4^*Y_2^*} - 2\sigma_{Y_4^*Y_1^*}\sigma_{Y_4^*Y_2^*} + \sigma^2_{Y_3^*Y_1^*}(8 - 2\sigma_{Y_4^*Y_1^*} + 3\sigma_{Y_4^*Y_2^*}) \\
&+ \sigma_{Y_2^*Y_1^*}\Big(-2 - 2\sigma^2_{Y_4^*Y_1^*} + 2\sigma_{Y_4^*Y_2^*} + \sigma_{Y_3^*Y_1^*}(-14 + 5\sigma_{Y_4^*Y_1^*} - 6\sigma_{Y_4^*Y_2^*} - 3\sigma_{Y_4^*Y_3^*}) + \sigma_{Y_4^*Y_1^*}(7 + 4\sigma_{Y_4^*Y_2^*} - 2\sigma_{Y_4^*Y_3^*}) - 7\sigma_{Y_4^*Y_3^*}\Big) \\
&+ 2\sigma_{Y_4^*Y_3^*} + \sigma_{Y_4^*Y_1^*}\sigma_{Y_4^*Y_3^*} + \sigma^2_{Y_2^*Y_1^*}(5 - 2\sigma_{Y_4^*Y_1^*} + 6\sigma_{Y_4^*Y_3^*}) + \sigma_{Y_3^*Y_1^*}\Big(\sigma^2_{Y_4^*Y_1^*} + \sigma_{Y_4^*Y_1^*}(-8 - 2\sigma_{Y_4^*Y_2^*} + \sigma_{Y_4^*Y_3^*}) + 2(2 + \sigma_{Y_4^*Y_2^*} + \sigma_{Y_4^*Y_3^*})\Big)\Big) \\
\sigma^2_{Y_5^*} &= -\frac{1}{(1 - 2\sigma_{Y_2^*Y_1^*} + \sigma_{Y_3^*Y_1^*})^6(-1 + 3\sigma_{Y_3^*Y_1^*} - 2\sigma_{Y_4^*Y_1^*})(2 - 3\sigma_{Y_2^*Y_1^*} + \sigma_{Y_4^*Y_1^*})}C^1
\end{aligned}$$

$$\begin{aligned}
\sigma_{Y_5^*Y_1^*} &= \frac{\sigma^2_{Y_2^*Y_1^*} + 3\sigma^2_{Y_3^*Y_1^*} + \sigma_{Y_4^*Y_1^*}(2+\sigma_{Y_4^*Y_1^*}) - 2\sigma_{Y_2^*Y_1^*}(\sigma_{Y_3^*Y_1^*}+\sigma_{Y_4^*Y_1^*}) - \sigma_{Y_3^*Y_1^*}(1+2\sigma_{Y_4^*Y_1^*})}{1-2\sigma_{Y_2^*Y_1^*}+\sigma_{Y_3^*Y_1^*}} \qquad (1)\\
\sigma_{Y_5^*Y_2^*} &= \frac{1}{(-1+2\sigma_{Y_2^*Y_1^*}-\sigma_{Y_3^*Y_1^*})(-1+3\sigma_{Y_3^*Y_1^*}-2\sigma_{Y_4^*Y_1^*})}\Big(\sigma^3_{Y_2^*Y_1^*} - \sigma_{Y_3^*Y_2^*} + \sigma_{Y_4^*Y_1^*} - 2\sigma_{Y_3^*Y_2^*}\sigma_{Y_4^*Y_1^*} - 3\sigma_{Y_3^*Y_2^*}\sigma^2_{Y_4^*Y_1^*} + 2\sigma_{Y_4^*Y_2^*} + 4\sigma_{Y_4^*Y_1^*}\sigma_{Y_4^*Y_2^*} + 2\sigma^2_{Y_4^*Y_1^*}\sigma_{Y_4^*Y_2^*}\\
&+ \sigma^2_{Y_3^*Y_1^*}(-2-9\sigma_{Y_3^*Y_2^*}+2\sigma_{Y_4^*Y_2^*}) + \sigma_{Y_3^*Y_1^*}(-2+\sigma_{Y_4^*Y_1^*}+2\sigma_{Y_3^*Y_2^*}(1+5\sigma_{Y_4^*Y_1^*}) - 4\sigma_{Y_4^*Y_2^*} - 4\sigma_{Y_4^*Y_1^*}\sigma_{Y_4^*Y_2^*}) + \sigma^2_{Y_2^*Y_1^*}(-4\sigma_{Y_3^*Y_1^*} - 3\sigma_{Y_3^*Y_2^*} + 2(-1+\sigma_{Y_4^*Y_1^*}+\sigma_{Y_4^*Y_2^*}))\\
&+ \sigma_{Y_2^*Y_1^*}\Big(1+4\sigma^2_{Y_3^*Y_1^*} - 2\sigma_{Y_3^*Y_2^*}(-1+\sigma_{Y_4^*Y_1^*}) - 2\sigma_{Y_4^*Y_1^*} + \sigma^2_{Y_4^*Y_1^*} - 4\sigma_{Y_4^*Y_2^*} - 4\sigma_{Y_4^*Y_1^*}\sigma_{Y_4^*Y_2^*} + \sigma_{Y_3^*Y_1^*}(5+6\sigma_{Y_3^*Y_2^*} - 4\sigma_{Y_4^*Y_1^*} + 4\sigma_{Y_4^*Y_2^*})\Big)\Big)\\
\sigma_{Y_5^*Y_3^*} &= \frac{1}{(-1+2\sigma_{Y_2^*Y_1^*}-\sigma_{Y_3^*Y_1^*})(-2+3\sigma_{Y_2^*Y_1^*}-\sigma_{Y_4^*Y_1^*})}\Big(6\sigma^3_{Y_3^*Y_1^*} - \sigma_{Y_3^*Y_2^*} - 2\sigma_{Y_4^*Y_1^*} - 2\sigma_{Y_3^*Y_2^*}\sigma_{Y_4^*Y_1^*} + 2\sigma^2_{Y_4^*Y_1^*} - 3\sigma_{Y_3^*Y_2^*}\sigma^2_{Y_4^*Y_1^*} + 3\sigma_{Y_4^*Y_3^*} + 2\sigma_{Y_4^*Y_1^*}\sigma_{Y_4^*Y_3^*} + \sigma^2_{Y_4^*Y_1^*}\sigma_{Y_4^*Y_3^*}\\
&+ \sigma^2_{Y_3^*Y_1^*}(10-9\sigma_{Y_3^*Y_2^*}-7\sigma_{Y_4^*Y_1^*}+3\sigma_{Y_4^*Y_3^*}) + \sigma^2_{Y_2^*Y_1^*}(6-3\sigma_{Y_3^*Y_2^*}+9\sigma_{Y_4^*Y_3^*}) + \sigma_{Y_3^*Y_1^*}\Big(4-9\sigma_{Y_4^*Y_1^*}+2\sigma^2_{Y_4^*Y_1^*}+2\sigma_{Y_3^*Y_2^*}(1+5\sigma_{Y_4^*Y_1^*}) + 2\sigma_{Y_4^*Y_3^*} - 2\sigma_{Y_4^*Y_1^*}\sigma_{Y_4^*Y_3^*}\Big)\\
&- \sigma_{Y_2^*Y_1^*}\Big(3\sigma^2_{Y_3^*Y_1^*} + 2(1+\sigma_{Y_3^*Y_2^*}(-1+\sigma_{Y_4^*Y_1^*}) + \sigma_{Y_4^*Y_1^*}(-4+\sigma_{Y_4^*Y_3^*}) + 5\sigma_{Y_4^*Y_3^*}) + \sigma_{Y_3^*Y_1^*}(17-6\sigma_{Y_3^*Y_2^*}-2\sigma_{Y_4^*Y_1^*}+6\sigma_{Y_4^*Y_3^*})\Big)\Big)\\
\sigma_{Y_5^*Y_4^*} &= -\frac{1}{(1-2\sigma_{Y_2^*Y_1^*}+\sigma_{Y_3^*Y_1^*})^3}\Big(2\sigma_{Y_4^*Y_1^*} - 2\sigma^2_{Y_4^*Y_1^*} - 2\sigma^3_{Y_4^*Y_1^*} + 2\sigma_{Y_4^*Y_2^*} + 4\sigma_{Y_4^*Y_1^*}\sigma_{Y_4^*Y_2^*} + 2\sigma^2_{Y_4^*Y_1^*}\sigma_{Y_4^*Y_2^*} + \sigma^2_{Y_2^*Y_1^*}\Big(-8-4\sigma^2_{Y_4^*Y_1^*}+10\sigma_{Y_4^*Y_2^*}\\
&+ 3\sigma_{Y_3^*Y_1^*}(-6+5\sigma_{Y_4^*Y_1^*}-2\sigma_{Y_4^*Y_2^*}-7\sigma_{Y_4^*Y_3^*}) + 4\sigma_{Y_4^*Y_1^*}(3+2\sigma_{Y_4^*Y_2^*}-\sigma_{Y_4^*Y_3^*}) - 29\sigma_{Y_4^*Y_3^*}\Big) + \sigma^3_{Y_3^*Y_1^*}(4+2\sigma_{Y_4^*Y_2^*}-3\sigma_{Y_4^*Y_3^*}) - 3\sigma_{Y_4^*Y_3^*} - 2\sigma_{Y_4^*Y_1^*}\sigma_{Y_4^*Y_3^*} - \sigma^2_{Y_4^*Y_1^*}\sigma_{Y_4^*Y_3^*}\\
&+ \sigma^3_{Y_2^*Y_1^*}(7-6\sigma_{Y_4^*Y_1^*}-4\sigma_{Y_4^*Y_2^*}+18\sigma_{Y_4^*Y_3^*}) - \sigma_{Y_3^*Y_1^*}\Big(4-8\sigma_{Y_4^*Y_1^*}+\sigma^3_{Y_4^*Y_1^*}+2\sigma_{Y_4^*Y_2^*}+5\sigma_{Y_4^*Y_3^*}+\sigma^2_{Y_4^*Y_1^*}(-6-2\sigma_{Y_4^*Y_2^*}+\sigma_{Y_4^*Y_3^*})\Big) + \sigma^2_{Y_3^*Y_1^*}\Big(-8+2\sigma^2_{Y_4^*Y_1^*}-2\sigma_{Y_4^*Y_2^*}-5\sigma_{Y_4^*Y_3^*}\\
&+ \sigma_{Y_4^*Y_1^*}(-6-4\sigma_{Y_4^*Y_2^*}+2\sigma_{Y_4^*Y_3^*})\Big) + \sigma_{Y_2^*Y_1^*}\Big(2+2\sigma^3_{Y_4^*Y_1^*}-8\sigma_{Y_4^*Y_2^*}-2\sigma_{Y_4^*Y_1^*}(5+6\sigma_{Y_4^*Y_2^*}-3\sigma_{Y_4^*Y_3^*}) - 6\sigma^2_{Y_3^*Y_1^*}(-1+\sigma_{Y_4^*Y_1^*}-2\sigma_{Y_4^*Y_3^*}) + 16\sigma_{Y_4^*Y_3^*} + \sigma^2_{Y_4^*Y_1^*}(3-4\sigma_{Y_4^*Y_2^*}+2\sigma_{Y_4^*Y_3^*})\\
&- 2\sigma_{Y_3^*Y_1^*}\Big(\sigma^2_{Y_4^*Y_1^*}+\sigma_{Y_4^*Y_1^*}(6-2\sigma_{Y_4^*Y_2^*}+\sigma_{Y_4^*Y_3^*}) - 2(5+2\sigma_{Y_4^*Y_2^*}+5\sigma_{Y_4^*Y_3^*})\Big)\Big)\Big)
\end{aligned}$$

[1] C is a very complicated function of all the polychoric covariances

Alternative 1 parameterization

$$\sigma^2_{Y_2^*} = \frac{1}{\sigma^2_{Y_1^*} - 3\sigma_{Y_3^*Y_1^*} + 2\sigma_{Y_4^*Y_1^*}} \Big(\sigma^2_{Y_2^*Y_1^*} + \sigma_{Y_3^*Y_1^*} + \sigma_{Y_3^*Y_2^*}\sigma_{Y_4^*Y_1^*} + \sigma^2_{Y_1^*}(1 + 2\sigma_{Y_3^*Y_2^*} - \sigma_{Y_4^*Y_2^*}) - \sigma_{Y_3^*Y_1^*}\sigma_{Y_4^*Y_2^*} + \sigma_{Y_2^*Y_1^*}(-2 - 2\sigma_{Y_3^*Y_1^*} - 3\sigma_{Y_3^*Y_2^*} + \sigma_{Y_4^*Y_1^*} + 2\sigma_{Y_4^*Y_2^*})\Big)$$

$$\sigma_{Y_5^*Y_1^*} = \frac{\sigma^2_{Y_2^*Y_1^*} - \sigma^2_{Y_1^*}\sigma_{Y_3^*Y_1^*} + 3\sigma^2_{Y_3^*Y_1^*} + 2\sigma^2_{Y_1^*}\sigma_{Y_4^*Y_1^*} - 2\sigma_{Y_3^*Y_1^*}\sigma_{Y_4^*Y_1^*} + \sigma^2_{Y_4^*Y_1^*} - 2\sigma_{Y_2^*Y_1^*}(\sigma_{Y_3^*Y_1^*} + \sigma_{Y_4^*Y_1^*})}{\sigma^2_{Y_1^*} - 2\sigma_{Y_2^*Y_1^*} + \sigma_{Y_3^*Y_1^*}}$$

$$\begin{aligned}
\sigma_{Y_5^*Y_2^*} &= \frac{1}{(\sigma^2_{Y_1^*} - 2\sigma_{Y_2^*Y_1^*} + \sigma_{Y_3^*Y_1^*})(\sigma^2_{Y_1^*} - 3\sigma_{Y_3^*Y_1^*} + 2\sigma_{Y_4^*Y_1^*})} \Big(\sigma^3_{Y_2^*Y_1^*} - 2\sigma^2_{Y_3^*Y_1^*} - 9\sigma^2_{Y_3^*Y_1^*}\sigma_{Y_3^*Y_2^*} + \sigma_{Y_3^*Y_1^*}\sigma_{Y_4^*Y_1^*} + 10\sigma_{Y_3^*Y_1^*}\sigma_{Y_3^*Y_2^*}\sigma_{Y_4^*Y_1^*} - 3\sigma_{Y_3^*Y_2^*}\sigma^2_{Y_4^*Y_1^*} - \sigma^2_{Y_1^*}(\sigma_{Y_3^*Y_2^*} - 2\sigma_{Y_4^*Y_2^*}) \\
&+ 2\sigma^2_{Y_3^*Y_1^*}\sigma_{Y_4^*Y_2^*} - 4\sigma_{Y_3^*Y_1^*}\sigma_{Y_4^*Y_1^*}\sigma_{Y_4^*Y_2^*} + 2\sigma^2_{Y_4^*Y_1^*}\sigma_{Y_4^*Y_2^*} + \sigma^2_{Y_1^*}(\sigma_{Y_4^*Y_1^*} - 2\sigma_{Y_3^*Y_2^*}\sigma_{Y_4^*Y_1^*} + \sigma_{Y_2^*Y_1^*}(1 + 2\sigma_{Y_3^*Y_2^*} - 4\sigma_{Y_4^*Y_2^*}) + 2\sigma_{Y_3^*Y_1^*}(-1 + \sigma_{Y_3^*Y_2^*} - 2\sigma_{Y_4^*Y_2^*}) + 4\sigma_{Y_4^*Y_1^*}\sigma_{Y_4^*Y_2^*}) \\
&+ \sigma^2_{Y_2^*Y_1^*}(-4\sigma_{Y_3^*Y_1^*} - 3\sigma_{Y_3^*Y_2^*} + 2(-1 + \sigma_{Y_4^*Y_1^*} + \sigma_{Y_4^*Y_2^*})) + \sigma_{Y_2^*Y_1^*}\Big(4\sigma^2_{Y_3^*Y_1^*} + \sigma_{Y_4^*Y_1^*}(-2 - 2\sigma_{Y_3^*Y_2^*} + \sigma_{Y_4^*Y_1^*} - 4\sigma_{Y_4^*Y_2^*}) + \sigma_{Y_3^*Y_1^*}(5 + 6\sigma_{Y_3^*Y_2^*} - 4\sigma_{Y_4^*Y_1^*} + 4\sigma_{Y_4^*Y_2^*})\Big)\Big)
\end{aligned}$$

$$\begin{aligned}
\sigma_{Y_5^*Y_3^*} &= \frac{1}{2}\Bigg(-\frac{(\sigma_{Y_2^*Y_1^*} - 2\sigma_{Y_3^*Y_1^*} + \sigma_{Y_4^*Y_1^*})}{\sigma^2_{Y_1^*} - 2\sigma_{Y_2^*Y_1^*} + \sigma_{Y_3^*Y_1^*}} - \frac{3\sigma^2_{Y_3^*}(\sigma_{Y_2^*Y_1^*} - 2\sigma_{Y_3^*Y_1^*} + \sigma_{Y_4^*Y_1^*})}{\sigma^2_{Y_1^*} - 2\sigma_{Y_2^*Y_1^*} + \sigma_{Y_3^*Y_1^*}} + \frac{(\sigma_{Y_2^*Y_1^*} - 2\sigma_{Y_3^*Y_1^*} + \sigma_{Y_4^*Y_1^*})^2\Big(2\sigma^2_{Y_2^*Y_1^*} - \sigma_{Y_2^*Y_1^*}\sigma_{Y_3^*Y_1^*} + \sigma^2_{Y_1^*}(-2\sigma_{Y_3^*Y_1^*} + \sigma_{Y_4^*Y_1^*})\Big)}{(\sigma^2_{Y_1^*} - 2\sigma_{Y_2^*Y_1^*} + \sigma_{Y_3^*Y_1^*})^3} \\
&+ \frac{1}{(\sigma^2_{Y_1^*} - 2\sigma_{Y_2^*Y_1^*} + \sigma_{Y_3^*Y_1^*})^2(\sigma^2_{Y_1^*} - 3\sigma_{Y_3^*Y_1^*} + 2\sigma_{Y_4^*Y_1^*})}\Big((\sigma^2_{Y_1^*} - 2\sigma_{Y_2^*Y_1^*} + \sigma_{Y_3^*Y_1^*})^3\sigma_{Y_3^*Y_2^*} - (\sigma^2_{Y_1^*} - 2\sigma_{Y_2^*Y_1^*} + \sigma_{Y_3^*Y_1^*})^2(\sigma_{Y_2^*Y_1^*} - 2\sigma_{Y_3^*Y_1^*} + \sigma_{Y_4^*Y_1^*}) \\
&+ (\sigma^2_{Y_1^*} - 2\sigma_{Y_2^*Y_1^*} + \sigma_{Y_3^*Y_1^*})^2\sigma_{Y_3^*Y_2^*}(\sigma_{Y_2^*Y_1^*} - 2\sigma_{Y_3^*Y_1^*} + \sigma_{Y_4^*Y_1^*}) + (\sigma^2_{Y_1^*} - 2\sigma_{Y_2^*Y_1^*} + \sigma_{Y_3^*Y_1^*})\sigma_{Y_3^*Y_2^*}(\sigma_{Y_2^*Y_1^*} - 2\sigma_{Y_3^*Y_1^*} + \sigma_{Y_4^*Y_1^*})^2 - \sigma^2_{Y_1^*}(\sigma_{Y_2^*Y_1^*} - 2\sigma_{Y_3^*Y_1^*} + \sigma_{Y_4^*Y_1^*})^3 \\
&+ (\sigma_{Y_2^*Y_1^*} - 2\sigma_{Y_3^*Y_1^*} + \sigma_{Y_4^*Y_1^*})^2\Big(2\sigma^2_{Y_2^*Y_1^*} - \sigma_{Y_2^*Y_1^*}\sigma_{Y_3^*Y_1^*} + \sigma^2_{Y_1^*}(-2\sigma_{Y_3^*Y_1^*} + \sigma_{Y_4^*Y_1^*})\Big) + (\sigma^2_{Y_1^*} - 2\sigma_{Y_2^*Y_1^*} + \sigma_{Y_3^*Y_1^*})(\sigma_{Y_2^*Y_1^*} - 2\sigma_{Y_3^*Y_1^*} + \sigma_{Y_4^*Y_1^*})\Big(\sigma^2_{Y_2^*Y_1^*} + \sigma^2_{Y_3^*Y_1^*} \\
&+ \sigma^2_{Y_1^*}(-\sigma_{Y_3^*Y_1^*} + \sigma_{Y_4^*Y_1^*}) - \sigma_{Y_2^*Y_1^*}(\sigma_{Y_3^*Y_1^*} + \sigma_{Y_4^*Y_1^*})\Big) + 2(\sigma_{Y_2^*Y_1^*} - 2\sigma_{Y_3^*Y_1^*} + \sigma_{Y_4^*Y_1^*})^2\Big(\sigma^2_{Y_2^*Y_1^*} + \sigma^2_{Y_3^*Y_1^*} + \sigma^2_{Y_1^*}(-\sigma_{Y_3^*Y_1^*} + \sigma_{Y_4^*Y_1^*}) - \sigma_{Y_2^*Y_1^*}(\sigma_{Y_3^*Y_1^*} + \sigma_{Y_4^*Y_1^*})\Big) \\
&- (\sigma^2_{Y_1^*} - 2\sigma_{Y_2^*Y_1^*} + \sigma_{Y_3^*Y_1^*})^3\sigma_{Y_4^*Y_2^*} - (\sigma^2_{Y_1^*} - 2\sigma_{Y_2^*Y_1^*} + \sigma_{Y_3^*Y_1^*})^2(\sigma_{Y_2^*Y_1^*} - 2\sigma_{Y_3^*Y_1^*} + \sigma_{Y_4^*Y_1^*})\sigma_{Y_4^*Y_2^*}\Big) + 3\sigma_{Y_4^*Y_3^*} + \frac{2(\sigma_{Y_2^*Y_1^*} - 2\sigma_{Y_3^*Y_1^*} + \sigma_{Y_4^*Y_1^*})\sigma_{Y_4^*Y_3^*}}{\sigma^2_{Y_1^*} - 2\sigma_{Y_2^*Y_1^*} + \sigma_{Y_3^*Y_1^*}}\Bigg)
\end{aligned}$$

$$\sigma_{Y_5^*Y_4^*} = \frac{1}{2(\sigma^2_{Y_1^*} - 2\sigma_{Y_2^*Y_1^*} + \sigma_{Y_3^*Y_1^*})^4(\sigma^2_{Y_1^*} - 3\sigma_{Y_3^*Y_1^*} + 2\sigma_{Y_4^*Y_1^*})} C_2 \, [2]$$

[2] C_2 is a very complicated function of the polychoric covariances

Alternative 2 parameterization

$$\sigma_{Y_5^*Y_1^*} = \frac{\sigma^2_{Y_1^*Y_2^*} - \sigma^2_{Y_1^*}\sigma_{Y_1^*Y_3^*} - 2\sigma_{Y_1^*Y_2^*}\sigma_{Y_1^*Y_3^*} + 3\sigma^2_{Y_1^*Y_3^*} + 2\sigma^2_{Y_1^*}\sigma_{Y_1^*Y_4^*} - 2\sigma_{Y_1^*Y_2^*}\sigma_{Y_1^*Y_4^*} - 2\sigma_{Y_1^*Y_3^*}\sigma_{Y_1^*Y_4^*} + \sigma^2_{Y_1^*Y_4^*}}{\sigma^2_{Y_1^*} - 2\sigma_{Y_1^*Y_2^*} + \sigma_{Y_1^*Y_3^*}}$$

$$\sigma_{Y_5^*Y_2^*} = \frac{-\sigma^2_{Y_1^*}\sigma_{Y_2^*Y_3^*} + 3\sigma_{Y_1^*Y_3^*}\sigma_{Y_2^*Y_3^*} - 2\sigma_{Y_2^*Y_3^*}\sigma_{Y_1^*Y_4^*} + \sigma^2_{Y_2^*}(\sigma_{Y_1^*Y_2^*} - 2\sigma_{Y_1^*Y_3^*} + \sigma_{Y_1^*Y_4^*}) + 2\sigma^2_{Y_1^*}\sigma_{Y_2^*Y_4^*} - 3\sigma_{Y_1^*Y_2^*}\sigma_{Y_2^*Y_4^*} + \sigma_{Y_1^*Y_4^*}\sigma_{Y_2^*Y_4^*}}{\sigma^2_{Y_1^*} - 2\sigma_{Y_1^*Y_2^*} + \sigma_{Y_1^*Y_3^*}}$$

$$\begin{aligned}
\sigma_{Y_5^*Y_3^*} &= -\frac{1}{2(\sigma^2_{Y_1^*} - 2\sigma_{Y_1^*Y_2^*} + \sigma_{Y_1^*Y_3^*})^2}\Big(\sigma^3_{Y_1^*Y_2^*} - 6\sigma^2_{Y_2^*}\sigma^2_{Y_1^*Y_3^*} - 6\sigma^2_{Y_3^*}\sigma^2_{Y_1^*Y_3^*} + 2\sigma^3_{Y_1^*Y_3^*} + \sigma^2_{Y_1^*Y_3^*}\sigma_{Y_2^*Y_3^*} + \sigma^2_{Y_1^*Y_3^*}\sigma^2_{Y_4^*} + 7\sigma^2_{Y_2^*}\sigma_{Y_1^*Y_3^*}\sigma_{Y_1^*Y_4^*} + 3\sigma^2_{Y_3^*}\sigma_{Y_1^*Y_3^*}\sigma_{Y_1^*Y_4^*} - \sigma^2_{Y_1^*Y_3^*}\sigma_{Y_1^*Y_4^*} \\
&- 2\sigma_{Y_1^*Y_3^*}\sigma_{Y_2^*Y_3^*}\sigma_{Y_1^*Y_4^*} - \sigma_{Y_1^*Y_3^*}\sigma^2_{Y_4^*}\sigma_{Y_1^*Y_4^*} - 2\sigma^2_{Y_2^*}\sigma^2_{Y_1^*Y_4^*} + \sigma_{Y_2^*Y_3^*}\sigma^2_{Y_1^*Y_4^*} + \sigma^2_{Y_1^*Y_3^*}\sigma_{Y_2^*Y_4^*} + \sigma^2_{Y_1^*}(\sigma_{Y_2^*Y_3^*} - \sigma^2_{Y_4^*} + \sigma_{Y_2^*Y_4^*} - 3\sigma_{Y_3^*Y_4^*}) + \sigma^2_{Y_1^*Y_3^*}\sigma_{Y_3^*Y_4^*} - 2\sigma_{Y_1^*Y_3^*}\sigma_{Y_1^*Y_4^*}\sigma_{Y_3^*Y_4^*} \\
&- \sigma^2_{Y_1^*Y_2^*}(6\sigma^2_{Y_3^*} + 2\sigma_{Y_1^*Y_3^*} - \sigma_{Y_2^*Y_3^*} + 2\sigma^2_{Y_4^*} - 2\sigma_{Y_1^*Y_4^*} - 4\sigma_{Y_2^*Y_4^*} + 8\sigma_{Y_3^*Y_4^*}) - \sigma^2_{Y_1^*}\Big(6\sigma^2_{Y_3^*}\sigma_{Y_1^*Y_3^*} - 2\sigma^2_{Y_1^*Y_3^*} + 2\sigma_{Y_1^*Y_3^*}\sigma_{Y_2^*Y_3^*} - 3\sigma^2_{Y_3^*}\sigma_{Y_1^*Y_4^*} + \sigma_{Y_1^*Y_3^*}\sigma_{Y_1^*Y_4^*} - 2\sigma_{Y_2^*Y_3^*}\sigma_{Y_1^*Y_4^*} \\
&+ \sigma^2_{Y_4^*}\sigma_{Y_1^*Y_4^*} + \sigma^2_{Y_2^*}(\sigma_{Y_1^*Y_2^*} - 2\sigma_{Y_1^*Y_3^*} + \sigma_{Y_1^*Y_4^*}) - 2\sigma_{Y_1^*Y_3^*}\sigma_{Y_2^*Y_4^*} + \sigma_{Y_1^*Y_2^*}(-3\sigma^2_{Y_3^*} + \sigma_{Y_1^*Y_3^*} + 2\sigma_{Y_2^*Y_3^*} - 3\sigma^2_{Y_4^*} + 4\sigma_{Y_2^*Y_4^*} - 10\sigma_{Y_3^*Y_4^*}) + 2\sigma_{Y_1^*Y_3^*}\sigma_{Y_3^*Y_4^*} + 2\sigma_{Y_1^*Y_4^*}\sigma_{Y_3^*Y_4^*}\Big) \\
&+ \sigma_{Y_1^*Y_2^*}\Big(3\sigma^2_{Y_2^*}\sigma_{Y_1^*Y_3^*} + 15\sigma^2_{Y_3^*}\sigma_{Y_1^*Y_3^*} - \sigma^2_{Y_1^*Y_3^*} + 2\sigma_{Y_1^*Y_3^*}\sigma_{Y_2^*Y_3^*} - \sigma_{Y_1^*Y_3^*}\sigma^2_{Y_4^*} - 2\sigma^2_{Y_2^*}\sigma_{Y_1^*Y_4^*} - 6\sigma^2_{Y_3^*}\sigma_{Y_1^*Y_4^*} - 2\sigma_{Y_1^*Y_3^*}\sigma_{Y_1^*Y_4^*} - 2\sigma_{Y_2^*Y_3^*}\sigma_{Y_1^*Y_4^*} + 2\sigma^2_{Y_4^*}\sigma_{Y_1^*Y_4^*} + \sigma^2_{Y_1^*Y_4^*} \\
&- 4\sigma_{Y_1^*Y_3^*}\sigma_{Y_2^*Y_4^*} + 2\sigma_{Y_1^*Y_3^*}\sigma_{Y_3^*Y_4^*} + 4\sigma_{Y_1^*Y_4^*}\sigma_{Y_3^*Y_4^*}\Big)\Big)
\end{aligned}$$

$$\begin{aligned}
\sigma_{Y_5^*Y_4^*} &= \frac{1}{2(\sigma^2_{Y_1^*} - 2\sigma_{Y_1^*Y_2^*} + \sigma_{Y_1^*Y_3^*})^3}\Big(-\sigma^4_{Y_1^*Y_2^*} - 12\sigma^2_{Y_2^*}\sigma^3_{Y_1^*Y_3^*} - 12\sigma^4_{Y_1^*Y_3^*} + 18\sigma^3_{Y_1^*Y_3^*}\sigma_{Y_2^*Y_3^*} - 10\sigma^3_{Y_1^*Y_3^*}\sigma^2_{Y_4^*} + 20\sigma^2_{Y_2^*}\sigma^2_{Y_1^*Y_3^*}\sigma_{Y_1^*Y_4^*} + 6\sigma^2_{Y_3^*}\sigma^2_{Y_1^*Y_3^*}\sigma_{Y_1^*Y_4^*} + 4\sigma^3_{Y_1^*Y_3^*}\sigma_{Y_1^*Y_4^*} - 25\sigma^2_{Y_1^*Y_3^*}\sigma_{Y_2^*Y_3^*}\sigma_{Y_1^*Y_4^*} \\
&+ 5\sigma^2_{Y_1^*Y_3^*}\sigma^2_{Y_4^*}\sigma_{Y_1^*Y_4^*} - 11\sigma^2_{Y_2^*}\sigma_{Y_1^*Y_3^*}\sigma^2_{Y_1^*Y_4^*} - 3\sigma^2_{Y_3^*}\sigma_{Y_1^*Y_3^*}\sigma^2_{Y_1^*Y_4^*} + \sigma^2_{Y_1^*Y_3^*}\sigma^2_{Y_1^*Y_4^*} + 10\sigma_{Y_1^*Y_3^*}\sigma_{Y_2^*Y_3^*}\sigma^2_{Y_1^*Y_4^*} + \sigma_{Y_1^*Y_3^*}\sigma^2_{Y_4^*}\sigma^2_{Y_1^*Y_4^*} + 2\sigma^2_{Y_2^*}\sigma^3_{Y_1^*Y_4^*} - \sigma_{Y_2^*Y_3^*}\sigma^3_{Y_1^*Y_4^*} + 18\sigma^3_{Y_1^*Y_3^*}\sigma_{Y_2^*Y_4^*} \\
&- 13\sigma^2_{Y_1^*Y_3^*}\sigma_{Y_1^*Y_4^*}\sigma_{Y_2^*Y_4^*} + \sigma^3_{Y_1^*Y_2^*}(-18\sigma^2_{Y_3^*} + 12\sigma_{Y_1^*Y_3^*} + 3\sigma_{Y_2^*Y_3^*} + 2\sigma^2_{Y_4^*} + 13\sigma_{Y_1^*Y_4^*} + 12\sigma_{Y_2^*Y_4^*} - 36\sigma_{Y_3^*Y_4^*}) + 3\sigma^2_{Y_1^*Y_3^*}\sigma_{Y_1^*Y_4^*}\sigma_{Y_3^*Y_4^*} + {\sigma^2_{Y_1^*}}^3(4\sigma^2_{Y_4^*} - 8\sigma_{Y_2^*Y_4^*} + 6\sigma_{Y_3^*Y_4^*}) \\
&+ {\sigma^2_{Y_1^*}}^2\Big(12\sigma^2_{Y_3^*}\sigma_{Y_1^*Y_3^*} - 6\sigma_{Y_1^*Y_3^*}\sigma_{Y_2^*Y_3^*} - 6\sigma_{Y_1^*Y_3^*}\sigma^2_{Y_4^*} - 6\sigma^2_{Y_3^*}\sigma_{Y_1^*Y_4^*} - 8\sigma_{Y_1^*Y_3^*}\sigma_{Y_1^*Y_4^*} + 3\sigma_{Y_2^*Y_3^*}\sigma_{Y_1^*Y_4^*} + 9\sigma^2_{Y_4^*}\sigma_{Y_1^*Y_4^*} + 4\sigma^2_{Y_1^*Y_4^*} + 2\sigma_{Y_1^*Y_3^*}\sigma_{Y_2^*Y_4^*} - 13\sigma_{Y_1^*Y_4^*}\sigma_{Y_2^*Y_4^*} \\
&+ \sigma_{Y_1^*Y_2^*}(-6\sigma^2_{Y_3^*} + 3\sigma_{Y_2^*Y_3^*} - 15\sigma^2_{Y_4^*} + 4\sigma_{Y_1^*Y_4^*} + 35\sigma_{Y_2^*Y_4^*} - 33\sigma_{Y_3^*Y_4^*}) + 12\sigma_{Y_1^*Y_3^*}\sigma_{Y_3^*Y_4^*} + 3\sigma_{Y_1^*Y_4^*}\sigma_{Y_3^*Y_4^*}\Big) + \sigma^2_{Y_1^*Y_2^*}\Big(-3\sigma^2_{Y_2^*}\sigma_{Y_1^*Y_3^*} + 45\sigma^2_{Y_3^*}\sigma_{Y_1^*Y_3^*} - 39\sigma^2_{Y_1^*Y_3^*} + 6\sigma_{Y_1^*Y_3^*}\sigma_{Y_2^*Y_3^*} \\
&- 51\sigma_{Y_1^*Y_3^*}\sigma^2_{Y_4^*} + 2\sigma^2_{Y_2^*}\sigma_{Y_1^*Y_4^*} - 12\sigma^2_{Y_3^*}\sigma_{Y_1^*Y_4^*} - 24\sigma_{Y_1^*Y_3^*}\sigma_{Y_1^*Y_4^*} - 7\sigma_{Y_2^*Y_3^*}\sigma_{Y_1^*Y_4^*} + 32\sigma^2_{Y_4^*}\sigma_{Y_1^*Y_4^*} + 13\sigma^2_{Y_1^*Y_4^*} + 60\sigma_{Y_1^*Y_3^*}\sigma_{Y_2^*Y_4^*} - 52\sigma_{Y_1^*Y_4^*}\sigma_{Y_2^*Y_4^*} + 36\sigma_{Y_1^*Y_3^*}\sigma_{Y_3^*Y_4^*} + 12\sigma_{Y_1^*Y_4^*}\sigma_{Y_3^*Y_4^*}\Big) \\
&- \sigma_{Y_1^*Y_2^*}\Big(-44\sigma^3_{Y_1^*Y_3^*} + 33\sigma^2_{Y_1^*Y_3^*}\sigma_{Y_2^*Y_3^*} - 45\sigma^2_{Y_1^*Y_3^*}\sigma^2_{Y_4^*} + 6\sigma^2_{Y_1^*Y_3^*}\sigma_{Y_1^*Y_4^*} - 40\sigma_{Y_1^*Y_3^*}\sigma_{Y_2^*Y_3^*}\sigma_{Y_1^*Y_4^*} + 26\sigma_{Y_1^*Y_3^*}\sigma^2_{Y_4^*}\sigma_{Y_1^*Y_4^*} + 4\sigma_{Y_1^*Y_3^*}\sigma^2_{Y_1^*Y_4^*} + 11\sigma_{Y_2^*Y_3^*}\sigma^2_{Y_1^*Y_4^*} + 2\sigma^2_{Y_4^*}\sigma^2_{Y_1^*Y_4^*} + \sigma^3_{Y_1^*Y_4^*} \\
&+ 6\sigma^2_{Y_3^*}\Big(3\sigma^2_{Y_1^*Y_3^*} + \sigma_{Y_1^*Y_3^*}\sigma_{Y_1^*Y_4^*} - \sigma^2_{Y_1^*Y_4^*}\Big) - 2\sigma^2_{Y_2^*} + (6\sigma^2_{Y_1^*Y_3^*} - 7\sigma_{Y_1^*Y_3^*}\sigma_{Y_1^*Y_4^*} + 2\sigma^2_{Y_1^*Y_4^*}\Big) + 69\sigma^2_{Y_1^*Y_3^*}\sigma_{Y_2^*Y_4^*} - 52\sigma_{Y_1^*Y_3^*}\sigma_{Y_1^*Y_4^*}\sigma_{Y_2^*Y_4^*} + 9\sigma^2_{Y_1^*Y_3^*}\sigma_{Y_3^*Y_4^*} + 12\sigma_{Y_1^*Y_3^*}\sigma_{Y_1^*Y_4^*}\sigma_{Y_3^*Y_4^*}\Big) \\
&- \sigma^2_{Y_1^*}\Big(12\sigma^2_{Y_3^*}\sigma^2_{Y_1^*Y_3^*} - 12\sigma^3_{Y_1^*Y_3^*} + 4\sigma^2_{Y_1^*Y_3^*}\sigma_{Y_2^*Y_3^*} + 20\sigma^2_{Y_1^*Y_3^*}\sigma^2_{Y_4^*} - 4\sigma^2_{Y_1^*Y_3^*}\sigma_{Y_1^*Y_4^*} - 10\sigma_{Y_1^*Y_3^*}\sigma_{Y_2^*Y_3^*}\sigma_{Y_1^*Y_4^*} + 14\sigma_{Y_1^*Y_3^*}\sigma^2_{Y_4^*}\sigma_{Y_1^*Y_4^*} - 3\sigma^2_{Y_3^*}\sigma^2_{Y_1^*Y_4^*} + 5\sigma_{Y_1^*Y_3^*}\sigma^2_{Y_1^*Y_4^*} + 4\sigma_{Y_2^*Y_3^*}\sigma^2_{Y_1^*Y_4^*} + \\
&+ \sigma^2_{Y_4^*}\sigma^2_{Y_1^*Y_4^*} + \sigma^2_{Y_2^*}(\sigma_{Y_1^*Y_2^*} - 2\sigma_{Y_1^*Y_3^*} + \sigma_{Y_1^*Y_4^*})^2 + 28\sigma^2_{Y_1^*Y_3^*}\sigma_{Y_2^*Y_4^*} - 26\sigma_{Y_1^*Y_3^*}\sigma_{Y_1^*Y_4^*}\sigma_{Y_2^*Y_4^*} + 6\sigma^2_{Y_1^*Y_3^*}\sigma_{Y_3^*Y_4^*} + 6\sigma_{Y_1^*Y_3^*}\sigma_{Y_1^*Y_4^*}\sigma_{Y_3^*Y_4^*} + \sigma^2_{Y_1^*Y_2^*}(21\sigma^2_{Y_3^*} - 3\sigma_{Y_1^*Y_3^*} - 8\sigma_{Y_2^*Y_3^*} + 13\sigma^2_{Y_4^*} \\
&- 16\sigma_{Y_1^*Y_4^*} - 44\sigma_{Y_2^*Y_4^*} + 60\sigma_{Y_3^*Y_4^*}) - 2\sigma_{Y_1^*Y_2^*}\Big(-6\sigma^2_{Y_1^*Y_3^*} + 3\sigma^2_{Y_3^*}(8\sigma_{Y_1^*Y_3^*} - 3\sigma_{Y_1^*Y_4^*}) + \sigma_{Y_1^*Y_4^*}(2\sigma_{Y_2^*Y_3^*} + 17\sigma^2_{Y_4^*} + 8\sigma_{Y_1^*Y_4^*} - 26\sigma_{Y_2^*Y_4^*} + 6\sigma_{Y_3^*Y_4^*}) \\
&+ \sigma_{Y_1^*Y_3^*}(-7\sigma_{Y_2^*Y_3^*} - 19\sigma^2_{Y_4^*} - 17\sigma_{Y_1^*Y_4^*} + 17\sigma_{Y_2^*Y_4^*} + 21\sigma_{Y_3^*Y_4^*})\Big)\Big)\Big)
\end{aligned}$$
